# The translational revolution in atopic dermatitis: the paradigm shift from pathogenesis to treatment

**DOI:** 10.1038/s41423-023-00992-4

**Published:** 2023-03-16

**Authors:** Paola Facheris, Jane Jeffery, Ester Del Duca, Emma Guttman-Yassky

**Affiliations:** 1 Laboratory of Inflammatory Skin Diseases, Department of Dermatology, Icahn School of Medicine at Mount Sinai, New York, NY USA; 2 Humanitas Clinical and Research Center, Department of Dermatology, Rozzano, Milano, Italy; 3 Duke University School of Medicine, Durham, NC USA; 4 Department of Dermatology, Icahn School of Medicine at Mount Sinai, New York, NY USA

**Keywords:** Atopic dermatitis, translational revolution, biomarkers, eczema, Diagnostic markers, Predictive markers, Target identification, Interleukins

## Abstract

Atopic dermatitis (AD) is the most common inflammatory skin disease, and it is considered a complex and heterogeneous condition. Different phenotypes of AD, defined according to the patient age at onset, race, and ethnic background; disease duration; and other disease characteristics, have been recently described, underlying the need for a personalized treatment approach. Recent advancements in understanding AD pathogenesis resulted in a real translational revolution and led to the exponential expansion of the therapeutic pipeline. The study of biomarkers in clinical studies of emerging treatments is helping clarify the role of each cytokine and immune pathway in AD and will allow addressing the unique immune fingerprints of each AD subset. Personalized medicine will be the ultimate goal of this targeted translational research. In this review, we discuss the changes in the concepts of both the pathogenesis of and treatment approach to AD, highlight the scientific rationale behind each targeted treatment and report the most recent clinical efficacy data.

## Introduction

Atopic dermatitis (AD) is the most common inflammatory skin condition, affecting up to 25% of children and between 4 and 7% of adults [1]. An estimated 85% of AD cases appear before 5 years of age, but adult-onset AD is also common: 1 in 4 adults affected by AD in the US reports the onset of the disease in adulthood [2–4]. While most AD cases dissipate by adulthood, up to 33% of childhood-onset AD cases persist far into the life course [5]. Prevalence also varies based on ethnicity: African descendent individuals, as well as Asians and Pacific Islanders, are more likely to develop AD than Caucasian individuals [6–9].

AD lesions consist of oozing, pruritic, erythematous patches and papules, resulting in excoriation and serous exudate [2]. As the lesions become chronic, they turn dull, red, and lichenified. In the adult form, the classic AD locations include the symmetrical flexural regions, such as the antecubital and popliteal fosse. Other locations can be involved, including the face, head, neck, hands, trunk, and extensor surfaces of the limbs, with different manifestations based on the age at onset and acute vs. chronic disease [3, 10]. AD symptoms include daily pruritus and pain [11], sleep disturbances [12], and, in the most severe cases, depressive and anxiety-related symptoms [4, 12, 13]. Moreover, AD is considered the first manifestation of the so-called “atopic march”: other atopic conditions such as asthma, allergic rhinoconjunctivitis, and food allergy follow the appearance of AD in atopic predisposed patients [3, 10]. This multisystemic involvement heavily affects the quality of life of atopic individuals [11, 12]. In this review, we will cover the changes at the base of the paradigm shift we are experiencing in the treatment of AD. We will report the most recent translational research findings based on different specific pathogenetic axes, and we will review the impressive therapeutic pipeline for moderate-to-severe AD.

## The complex pathogenesis of AD: a current update

Two main contrasting hypotheses have been proposed in the past for the pathogenesis of AD. Based on the “outside-in” hypothesis, epidermal barrier dysfunction triggers immune activation; in contrast, based on the “inside-out” hypothesis, AD is primarily cytokine driven with secondary skin barrier dysfunction [14]. The modern approach to defining AD pathogenesis is now centered on integrating these two mechanisms and is oriented toward characterizing their interplay in AD [15].

Environmental noxious stimuli, immune dysregulation, genetic factors, impaired epidermal barrier integrity, and skin microbiome abnormalities all play pathogenetic roles in initiating and sustaining a state of chronic inflammation in AD and contribute to orchestrating the disease phenotype.

Epidermal barrier dysfunction in AD is characterized by a lower expression of terminal differentiation markers, such as filaggrin (FLG) and loricrin (LOR), and by a higher permeability defect caused by skin lipid film impairment and higher transepidermal water loss [16, 17]. This makes AD skin more prone to the penetration of external agents (antigens, allergens, pollution, etc.) that are harmful to keratinocytes. Damaged keratinocytes produce epidermal alarmins such as IL-33, IL-25, and TSLP, which activate the dendritic cells (DCs) and type 2 innate lymphoid cells (ILC2s) that produce IL-5 and IL-13, which activate eosinophils and Th2 cells [17]. Local Th2 polarization, in return, further diminishes barrier functions and sustains itching, causing skin barrier impairment and facilitating dysbiosis [16]. AD skin shows a higher proliferation of the members of the genus Staphylococcus, especially *S. aureus*, which can further damage keratinocytes and sustain local inflammation [18].

Pathogenic AD models have evolved similarly to those for psoriasis, linking specific T-cell subsets to different pathogenetic aspects of AD. In the past, several broad T-cell-targeting therapeutics (such as efalizumab, alefacept, phototherapy, and cyclosporine) showed efficacy in treating patients with moderate-to-severe AD [19–21]. TNF-alpha antagonists, such as infliximab [22] and etanercept [23], were ineffective in treating AD, suggesting the absence of a relevant role of TNF in AD pathogenesis. AD emerged as a prototypical Th2 disease, and this was supported by multiple observations, including increased levels of Th2 products and lower levels of IFN-γ in the blood of patients with severe AD [24, 25].

Subsequent studies highlighted that AD lesions are primarily, but not exclusively, Th2-driven, with the overproduction of important Th2 cytokines and chemokines, including IL-4, IL-5, IL-13, CCL17, CCL18, and CCL22 [25, 26]. but is also Th22-skewed, with the overproduction of IL-22, while the contributions of the Th1 and Th17 axes vary depending on the AD endophenotype [2, 23, 27, 28].

## AD phenotypes and endotypes

AD is no longer considered a homogeneous disease. Nevertheless, it encompasses a variety of endotypes and phenotypes based on the patient age at onset, race and ethnicity; disease chronicity; and IgE levels. On a molecular level, this characterization revealed specific immune pathway contributions and different characteristics of skin barrier alterations for each AD endophenotype [7, 9, 28–36].

Age at onset is an essential factor in determining AD endophenotypes. Neonatal AD shows low levels of Th1 and high levels of Th2 markers in the blood, which is associated with AD susceptibility [37–39]. The lesional skin of children with early-onset AD (at <6 months of age) shows strong Th2 upregulation and Th17/Th22 skewing but typically lacks Th1 activation, which is present in AD manifesting in adults [40]. In addition, Th9 expression is also higher in the lesional skin of pediatric patients with AD than in adult patients with AD [31]. Pediatric AD shows skin barrier defects consisting of epidermal hyperplasia with preserved FLG expression but a downregulation of tight junction and lipid barrier genes [31, 40]. AD in elderly patients demonstrates a lower level of Th2 and Th22 expression than AD in younger patients, with a parallel increase with age in Th1 and Th17 upregulation and a less pronounced barrier defect [41].

Several differences have been found according to disease duration. Acute AD, occurring within the first 72 h after lesion onset, is a predominantly Th2-driven disease process accompanied by an upregulation of the Th22 axis and increased AMP levels [25]. The chronic AD endotype features an upregulation of keratin 16 and Ki67, which is responsible for the characteristic hyperplasia of this stage of the disease [25]. Chronic AD features a continued increase in Th2 and Th22 expression and a new upregulation of the Th1 axis [2].

Ethnic background is a critical delineation for AD. European American AD cohorts feature relatively high activity of the Th2 and Th22 axes and some upregulation of the Th1 and Th17 axes compared to Asian and African American AD cohorts [28]. The Asian AD cohort tends to show the highest Th17 activation of any group studied and higher Th22 activation than European American cohorts [34, 42]. African American AD cohorts are characterized by an absence of Th17 and Th1 contributions with relatively low Th22 presence compared to other ethnicities studied [35].

Finally, AD can be classified into intrinsic and extrinsic forms. Extrinsic AD is primarily defined by its high levels of IgE in the serum as well as eosinophilia [43]. Extrinsic AD accounts for 80% of AD cases and is associated with greater rates of FLG mutations as well as a personal or family history of atopic disease [44]. Extrinsic AD severity correlates with increased Th2 axis expression and decreased barrier products, including FLG, LOR, and periplakin [45]. Intrinsic AD is phenotypically similar to extrinsic AD, but it is differentiated by female predominance, preserved barrier products, delayed disease onset, and an absence of a personal or family history of atopy [46, 47]. While sharing a similar Th2 signature, intrinsic AD is characterized by increased Th1 expression in the blood compared to extrinsic AD and lower CCL17 levels [48]. Intrinsic AD also shows greater cellular infiltrates with T cells, Langerhans cells, and myeloid DCs [33]. Th17- and Th22-driven expression of the antimicrobials S100A9 and S100A12 was found to be higher in intrinsic AD [49], with Th17 expression positively correlating with disease severity [48, 50].

## The translational revolution in AD

In the last decade, the AD therapeutic pipeline has been enriched, leading to new promising therapeutic perspectives. The key to this translational revolution has been the integration between bench studies of AD pathogenesis and the identification of biomarkers of therapeutic responses in clinical trials. This new approach led to the definition of the roles of the different cytokines and immune pathways involved in AD as well as to better disease stratification and therapeutic selection.

The first example of a successful translational model in AD is the one obtained with dupilumab, which led to a new era for the treatment of AD, offering a safe, long-lasting therapeutic option for patients with moderate-to-severe AD. Dupilumab, a monoclonal anti-IL-4Rα antibody, induces tissue reversal of the AD phenotype in the skin, improving both skin barrier function and immune dysregulation, and the molecular changes are parallel with clinical improvement [51, 52]. The most prominent effect is seen on the downregulation of Th2-associated chemokines, while the effect on the Th1 compartment is minimal [51]. Dupilumab treatment also reduces skin hyperplasia (K16 and ki67) and the inflammatory infiltrates of T cells and DCs [49]. Dupilumab was the first biologic therapy to be approved for the treatment of moderate-to-severe AD in adults (2017). The proven efficacy and the favorable safety profile led to subsequent extensions to patients 6 years or older (2021) and to patients 6 months and older (June 2022) [53].

Recently, other new effective treatments for AD have been approved by the FDA. After several successful phase III trials, in December 2021, tralokinumab was the first anti-IL-13 drug to be approved for adults with moderate-to-severe AD. In January 2022, two JAK inhibitors, abrocitinib and upadacitinib, received approval for moderate-to-severe AD in adults and in patients 12 years of age or older, respectively.

Although the AD therapeutic pipeline is rapidly expanding, the results of clinical trials have demonstrated that a considerable number of patients are unable to reach a clear or almost clear state with the currently available treatments. Dupilumab induces an Investigator Global Assessment (IGA) score of 0/1 in <40% of patients when administered as monotherapy or in combination with topical corticosteroids (TCSs) [54–57]. Less than 25% and <40% of tralokinumab-treated patients reach an IGA score if 0/1 when tralokinumab is used as monotherapy [58] or in combination with TCSs, respectively [59]. JAK inhibitors display higher rates of achieving IGA scores of 0/1, with upadacitinib able to clear ~60% of patients [60, 61] and abrocitinib <50% [62], with slightly higher rates when used in combination with TCSs [61, 63]. This lack of efficacy in certain patients highlights the need for other effective molecules and underlines the importance of developing targeted medicine to offer treatments tailored to patients’ characteristics.

**Fig. 1:**
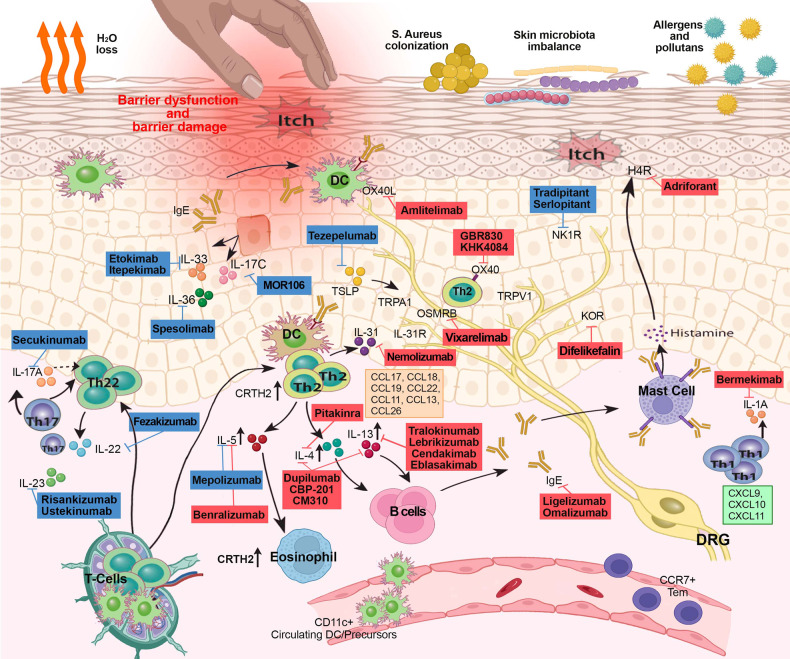
Schematic representation of the immune markers involved in the pathogenesis of AD and systemic drugs approved or that have been in clinical trial development for AD. In red boxes are reported molecules currently under investigation or with positive results in clinical trials, and in blue boxes are reported drugs that failed to reach primary endpoints in clinical trials. CRTH2 prostaglandin D2 receptor 2, DC dendritic cell, DRG dorsal root ganglion, H4R histamine H4 receptor, IL interleukin, KOR kappa opioid receptor, NK1R neurokinin1, OSM oncostatin M, OSMRB oncostatin M specific receptor subunit beta, OX40 tumor necrosis factor receptor superfamily, member 4, OX40L OX40 ligand, TSLP thymic stromal lymphoprotein, TRPA1 transient receptor potential cation channel, subfamily A, member 1, TRPV1 transient receptor potential vanilloid-1

Herein, we discuss the main pathways involved in AD pathogenesis and the corresponding treatment targets (Fig. 1). For each target, we summarize the most relevant results in the most advanced clinical trials in Tables 1–6.

**Table 1:** Systemic treatments for AD targeting the Th2 pathway

Axis	Target	Drug	Clinical trial (NCT number)	Primary endpoint	Results	Status of investigation
Th2	IL-4Rα	Dupilumab	NCT02277743 (LIBERTY AD SOLO 1, Phase III) and NCT02277769 (LIBERTY AD SOLO 2, Phase III).	IGA success at week 16.	SOLO 1: Primary endpoint occurred in 38% dupilumab Q2W vs. 37% dupilumab QW vs. 23% placebo. SOLO 2: The primary outcome occurred in 36%, 36 and 8% of the same corresponding groups, respectively [56].	FDA approved for moderate-to-severe AD in adults (2017) and children (6–11 years, 2021).
			NCT02260986 (LIBERTY AD CHRONOS, Phase III), combination with TCSs or TCI.	IGA success and achieving EASI75 at week 16.	IGA score of 0/1: 39% dupilumab Q2W vs. 39% dupilumab QW vs. 12% placebo) EASI75: 64% dupilumab Q2W vs. 69% dupilumab QW vs. 23% placebo [55].	
			NCT02755649 (LIBERTY AD CAFÉ, Phase III), combination with TCSs.	Achieving EASI75 at week 16.	Primary endpoint occurred in 62.6% dupilumab Q2W vs. 59.1% dupilumab QW and 29.6% placebo [54].	
		CBP-201	NCT04444752 (Phase II)	% Change in the EASI score at week 16.	% change in EASI score: −63.0% in 300 mg Q2W group vs. −65.4% in 300 mg Q4W group vs. −40.7% in placebo group [72].	Completed.
			NCT05017480 (Phase II)	IGA success at week 16.	Pending.	In progress.
		CM310	NCT04805411 (Phase II)	Achieving EASI75 at week 16.	EASI75 was achieved in 73.1% in the high-dose group vs. 70.6% in the low-dose group vs. 18.2% in the placebo group.	Phase III in progress (NCT05265923, NCT04893707).
	IL-4	Pitakinra	NCT00676884 (Phase II)	EASI score at Day 28.	Pending.	In progress.
	IL-13	Tralokinumab	NCT03131648 (ECZTRA 1, phase III), tralokinumab monotherapy. NCT03160885 (ECZTRA 2, phase III), tralokinumab monotherapy.	IGA success and achieving EASI75 at week 16.	ECZTRA 1, IGA score of 0/1: 15.8% tralokinumab Q2W vs. 7.1% placebo, EASI75:25% tralokinumab Q2W vs. 12.7% placebo. ECZTRA 2, IGA score of 0/1: 22.2 % tralokinumab Q2W vs. 10.8% placebo, EASI75: 33.2% tralokinumab Q2W vs. 11.4% placebo [58].	FDA approved for moderate-to-severe AD in adults (Dec 2021).
			NCT03363854 (ECZTRA 3, phase III), combination with TCSs.	IGA success and achieving EASI75 at week 16.	IGA score of 0/1: 38.9% tralokinumab Q2W vs. 26.2% placebo, EASI75: 56.0% tralokinumab Q2W vs. 35.7% placebo [59].	
			NCT04587453 (Japanese participants, phase III), combination with TCSs.	IGA success and achieving EASI75 at week 16.	Pending, study completed.	
			NCT03526861 (ECZTRA 6, Adolescent participants, phase III), combination with TCSs.	IGA success and achieving EASI75 at week 16.	IGA score of 0/1: 17.5% tralokinumab 300 mg Q2W, 21.4% tralokinumab 150 mg Q2W, 4.3% placebo. EASI 75:27.8% tralokinumab 300 mg Q2W, 28.6% tralokinumab 150 mg Q2W, 6.4% placebo [227].	
			NCT03761537 (ECZTRA 7, phase III), in combination with TCSs.	Achieving EASI75 at week 16.	EASI75: 64.2% tralokinumab Q2W vs. 50.5% placebo [74].	
			NCT03587805 (ECZTEND, phase III).	Number of adverse events.	Study is in progress.	
		Lebrikizumab	NCT04250337 (Adhere, phase III), combination with TCSs.	IGA success and achieving EASI75 at week 16.	IGA score of 0/1: 41% of patients receiving lebrikizumab vs. 22% placebo. EASI75: 70% lebrikizumab vs. 42% placebo [77].	Completed.
			NCT04146363 (ADvocate1, phase III), lebrikizumab monotherapy.	IGA success and achieving EASI75 at week 16.	IGA score of 0/1: 43% of lebrikizumab vs. 13% of placebo. EASI75: 59% of lebrikizumab vs. 16% of placebo [76].	
			NCT04178967 (ADvocate2, phase III), lebrikizumab monotherapy.	IGA success and achieving EASI75 at week 16.	IGA score of 0/1: 33% of lebrikizumab vs. 11% placebo. EASI75: 51% of lebrikizumab vs. 18% placebo [76].	
			NCT04250350 (ADore, phase III), adolescents.	% of participants discontinued from study treatment due to adverse events at week 52.	Still active.	In progress.
			NCT04760314 (Adhere-J, phase III), Japan.	IGA success and achieving EASI75 at week 16.	Still active.	In progress.
	IL-5	Mepolizumab	NCT03055195 (Phase II)	IGA success and achieving EASI75 at week 16.	Terminated for reaching futility criteria [84].	Terminated.
		Benralizumab	NCT03563066 (Phase II)	Number of eosinophils per mm2 of skin, 24 h after intradermal allergen challenge, at Day 65.	Pending.	Completed.
			NCT04605094 (Phase II)	IGA success at week 16.	Pending.	In progress.
	TSLP	Tezepelumab	NCT03809663 (Phase IIa)	IGA success at week 16.	IGA success rate was not significantly different in treatment group compared to control group [88].	Terminated.
	OX40L	Amlitelimab	NCT03754309 (Phase II)	% Change in the EASI score and the incidence of treatment-emergent adverse events (TEAEs).	% change in the EASI score: −80% in amlitelimab-LD group vs.−70% in amlitelimab-HD group vs. −49% in placebo group. Rate of TEAEs: 35% for amlitelimab-LD, 17% for amlitelimab-HD and 31% for placebo [95].	Completed.
			NCT05131477 (Phase II)	% Change in the EASI score from baseline to Day 113.	Pending.	In progress.
	OX40	GBR830	NCT02683928 (Phase II)	Achieving EASI50 at week 16.	EASI50 was achieved in 76.9% of GBR830-treated participants vs. 37.5% of placebo-treated participants [94].	Completed.
		KHK4083	NCT03703102 (Phase II)	% Change in the EASI score from baseline at week 16.	Submitted and awaiting review [98].	In progress.

The table summarizes the results of the most advanced clinical trials for each drug

*EASI* Eczema Area and Severity Index, *EASI 50* ≥50% reduction in the EASI score from baseline, *EASI 75* ≥75% reduction in the EASI score from baseline, *IGA* Investigator Global Assessment, IGA success, clear or almost clear (grade 0 or 1) and at least a two-grade improvement in the IGA score from baseline, *I-NRS* Itch Numerical Rating Scale, *QW* every week, *Q2W* every other week, *TCI* topical calcineurin-inhibitor, *TCS* topical corticosteroid

**Table 2:** Systemic treatments for AD targeting the Th17/IL-23 and Th22 pathways, the IL-1 family, IgE, and T-cell recruitment

Axis	Target	Drug	Clinical trial (NCT number)	Primary endpoint	Results	Status of investigation
Th17/ IL-23	IL-17A	Secukinumab	NCT03568136 (Phase II)	Achieving EASI50 at week 4.	Pending.	Completed.
			NCT02594098 (Phase II)	Fold change in epidermal thickness of lesional skin.	No significantly differences in epidermal thickness of lesional skin in secukinumab (1.18-fold for extrinsic AD, -1-fold for intrinsic AD) vs. placebo (1.15-fold for extrinsic AD, 1.5-fold for intrinsic AD). No significant changes in clinical scores (EASI and SCORAD) [106].	Terminated due to failure of primary endpoints.
	IL-12/23p40	Ustekinumab	NCT01945086 (Phase II)	% change in the EASI score at week 12.	No significant differences in 45 mg ustekinumab (−38.62%), 90 mg ustekinumab (−39.99%) compared to placebo (−37.54%) [102].	Completed.
			NCT01806662 (Phase II)	% Achieving SCORAD50 at week 16.	No significant differences in the treatment group (31.1%) compared to the placebo group (18.8%) [103].	Completed.
	IL-23p19	Risankizumab	NCT03706040 (Phase II)	% Achieving EASI75 at week 16.	No significant differences in risankizumab 150 mg (24.6%) and risankizumab 300 mg (21.7%) compared to placebo (11.8%) [104].	Completed.
Th22	IL-22	Fezakinumab/ ILV094	NCT01941537 (Phase II)	Decline in SCORAD at week 12.	Primary endpoint occurred in −13.8 + 2.7 fezakinumab vs. −8.0 + 3.1 placebo (nonsignificant, *p* = 0.134) in the entire population. In patients with severe AD (baseline SCORAD ≥ 50), the SCORAD decline was significantly stronger in the drug group vs. the placebo group (21.6 ± 3.8 vs. 9.6 ± 4.2, *p* = 0.029).	Completed.
	IL-17C	MOR106	NCT03864627 (Phase II)	Number of patients with TEAEs, Adverse events of special interest (AESIs), serious adverse events (SAEs) at Day 169.	Terminated for reaching futility criteria [116].	Terminated due to failure of primary endpoints.
IL-1 family	IL-1α	Bermekimab	NCT03496974 (Phase II)	Number of patients with TEAEs.	30% of patients receiving 200 mg of the drug had TEAEs, compared to 21.4% of patients receiving 400 mg of the drug [127].	Completed.
			NCT04021862 (Phase II)	Achieving EASI75 and TEAEs at week 16.	Pending.	Completed.
	IL-36	Spesolimab	NCT03822832 (Phase II)	% Change in the EASI score at week 16.	Pending.	Completed.
			NCT04086121 (Phase II)	Number of patients with TEAEs at week 48.	64.3% of participants had TEAEs at week 48 [128].	Completed.
	IL-33	Etokimab	NCT03533751 (ATLAS, Phase II)	% Change in the EASI score at week 16.	The study failed to meet the primary endpoint for each of the four etokimab dosing arms. Subsequent studies are currently postponed as data from the ATLAS trial are reevaluated [132].	Terminated.
		Itepekimab, REGN3500	NCT03736967 (Phase II) vs. dupilumab	% Change in the EASI score at week 16.	Terminated for lack of efficacy [133].	Terminated.
			NCT03738423 (Phase II)	% Change in the EASI score at week 16.	Pending.	Completed.
IgE	IgE	Omalizumab	NCT00367016 (Phase IV)	FcERI expression 3 months after drug administration.	FcERI expression was not significantly different between the group that received omalizumab (20.5 positive cells per field) and the control group (13.66 positive cells per field).	Completed.
			NCT02300701 (Phase IV, ADAPT trial, children, TCSs were allowed)	Decline in SCORAD at week 24.	The mean decline in SCORAD was −12.2 in omalizumab vs. −1.5 in placebo (*p* = 0.01) [161].	Completed.
		Ligelizumab	NCT01552629 (Phase II)	% Change in the EASI score at week 12.	Pending.	Completed.
T-cell recruitment	CCR4	RPT193	NCT04271514 (Phase I)	Incidence of TEAEs	Pending.	Completed.

The table summarizes the results of the most advanced clinical trials for each drug

*BSA* body surface area, *EASI* Eczema Area and Severity Index, *EASI 50* ≥50% reduction in the EASI score from baseline, *EASI 75* ≥75% reduction in the EASI score from baseline, *HD* high dose, *IGA* Investigator Global Assessment, IGA success, clear or almost clear (grade 0 or 1) and at least a two-grade improvement in the IGA score from baseline, *LD* low dose, *SCORAD* SCORing Atopic Dermatitis, *SCORAD50* ≥50% reduction in SCORAD from baseline

**Table 3:** Systemic treatments for AD targeting the JAK-STAT axis

Axis	Target	Drug	Clinical trial (NCT number)	Primary endpoint	Results	Status of investigation
JAK-STAT	JAK1	Abrocitinib	NCT03627767 (JADE REGIMEN, Phase III)	% of participants with loss of response during double blind period (week 40).	% loss of response: 77.5% (abrocitinib 200 mg OL to Placebo DB%), 39.6% (abrocitinib 200 mg OL to abrocitinib 100 mg + Placebo DB), 16.5% (abrocitinib 200 mg OL to abrocitinib 200 mg DB) [147].	FDA approved for the treatment of moderate-to-severe AD in adults (January 2022).
			NCT03796676 (JADE TEEN, phase III). In combination with TCSs in adolescents	IGA success and EASI75 at week 12.	IGA success: 46.2% abrocitinib 200 mg vs. 41.6% abrocitinib 100 mg vs. 24.5% placebo. EASI75: 71.0% abrocitinib 200 mg vs. 68.5% abrocitinib 100 mg vs. 41.5% placebo [61].	
			NCT03349060 (JADE-MONO1, Phase III) adult and adolescents.	IGA success and EASI75 at week 12.	IGA success: 44% abrocitinib 200 mg vs. 24% abrocitinib 100 mg vs. 8% placebo. EASI75: 63% abrocitinib 200 mg vs. 40% abrocitinib 100 mg) vs. 12% vs. placebo [62].	
			NCT03575871 ((JADE-MONO2, Phase III) adult and adolescents.	IGA success and EASI75 at week 12.	IGA success: 38.1% abrocitinib 200 mg vs. 28.4% abrocitinib 100 mg vs. 9.1% placebo. EASI75: 61% abrocitinib 200 mg vs. 44.5% abrocitinib 100 mg vs. 10.4% placebo [228].	
			NCT03720470 (JADE COMPARE, phase III) vs. dupilumab, in combination with topical therapy.	IGA success and EASI75 at week 12.	IGA success: 48.1% abrocitinib 200 mg vs. 36.6% abrocitinib 100 mg vs. 36.5% dupilumab vs. 14% placebo. EASI75: 70.3% abrocitinib 200 mg vs. 58.7% abrocitinib 100 mg vs. 58.1% dupilumab vs. 27.1% placebo [146, 228].	
			NCT04345367 (Phase III) vs. dupilumab, in combination with topical therapy.	Change in PP-NRS at week 2 and EASI90 at week 4.	Pending.	
		Upadacitinib	NCT03569293 (Measure Up 1, Phase III) adolescents and adults.	IGA success and achieving EASI75 at week 16.	IGA success: 62% upadacitinib 30 mg vs. 48% upadacitinib 15 mg vs. 8% vs. placebo. EASI75: 72.9% upadacitinib 30 mg vs. 60.1% upadacitinib 15 mg vs. 13.3% placebo[60].	FDA approved for the treatment of moderate-to-severe AD in patients 12 years of age and older (January 2022).
			NCT03607422 (Measure Up 2, Phase III) adolescents and adults.	IGA success and achieving EASI75 at week 16.	IGA success: 52% upadacitinib 30 mg vs. 39% upadacitinib 15 mg vs. 5% placebo. EASI75: 79.7% upadacitinib 30 mg vs. 69.6% upadacitinib 15 mg vs. 16.3% placebo [60].	
			NCT03568318 (Ad Up, Phase III) adolescents and adults, in combination with TCSs.	IGA success and achieving EASI75 at week 16.	IGA success: 59% upadacitinib 30 mg vs. 40% upadacitinib 15 mg vs. 11% placebo. EASI75: 77.1% upadacitinib 30 mg vs. 64.6% upadacitinib 15 mg vs. 26.4% placebo [63].	
			NCT03661138 (Rising Up, Phase III), adolescents and adults, in combination with TCSs.	Number (%) of participants experiencing adverse events at week 24.	56% in upadacitinib 15 mg; 64% in upadacitinib 30 mg, vs. 42% in placebo group. Acne, herpes zoster infections, anemia, neutropenia, CPK elevations were more frequent in the upadacitinib group [229].	
			NCT03738397 (Heads Up, Phase III) vs. dupilumab.	IGA success and achieving EASI75 at week 16.	EASI75: 71% upadacitinib 30 mg vs. 61.1% dupilumab [148].	
	JAK1/2	Baricitinib	NCT03334396 (BREEZE-AD1, Phase III)	IGA success and EASI75 week 16.	IGA success: 16.8% baricitinib 4 mg vs. 11.4% baricitinib 2 mg vs. 4.8% placebo [230].	Approved in Europe and Japan for the treatment of moderate-to-severe AD in adults.
			NCT03334422 (BREEZE-AD2, Phase III)	IGA success and achieving EASI75 at week 16.	IGA score of 0/1 success: 13.8% baricitinib 4 mg vs. 10.6% baricitinib 2 mg vs. 4.5% placebo [230].	
			NCT03334435 (BREEZE-AD3, Phase III)	IGA score of 0/1 at week 16, 36, 52.	Week 16: 45.7% (baricitinib 4 mg) vs. 46.3% (baricitinib 2 mg); week 68: 47.1% (baricitinib 4 mg) vs. 59.3% (baricitinib 2 mg) [231].	
			NCT03428100 (BREEZE-AD4, Phase III) in combination with TCSs.	Achieving EASI75 at week 16.	EASI75: 31.5% baricitinib 4 mg vs. 27.6% baricitinib 2 mg vs. 17.2% placebo. Only 4 mg was significant compared to placebo [232].	
			NCT03435081 (BREEZE-AD5, Phase III)	Achieving EASI75 at week 16.	EASI75: 29.5% baricitinib 2 mg, 12.9% baricitinib 1 mg vs. 8.2% placebo [149].	
			NCT03559270 (BREEZE-AD6, Phase III)	Achieving EASI75 at week 16.	Pending [233].	
			NCT03733301 (BREEZE-AD7, Phase III) in combination with TCSs.	IGA success and achieving EASI75 at week 16.	IGA success: 30.6% baricitinib 4 mg, 23.9% baricitinib 2 mg, 14.7% placebo [234].	
	JAK/SYK	Gusacitinib (ASN002)	NCT03531957 (RADIANT, Phase II)	% Change in the EASI score at week 12.	Submitted and awaiting QC approval [235].	Completed.
			NCT03654755 (Phase II)	Number and rate of TEAEs at week 104.	Terminated [236].	Terminated.

The table summarizes the results of the most advanced clinical trials for each drug

*CPKs* creatine phosphokinases, *EASI 75* ≥75% reduction in the EASI score from baseline, *IGA* Investigator Global Assessment, *IGA success* clear or almost clear (grade 0 or 1) and at least a two-grade improvement in the IGA score from baseline, *OL* open-label period, *PP-NRS* Peak Pruritus Numerical Rating Scale, *TEAEs* treatment-associated adverse events, *QC* quality control, *TCS* topical corticosteroid

**Table 4:** Systemic treatments for AD targeting PDE, arachidonic acid, and pruritus axes

Axis	Target	Drug	Clinical trial (NCT number)	Primary endpoint	Results	Status of investigation
PDE	PDE4	Apremilast	NCT01393158 (Phase II, pilot study), no placebo arm included.	% Change in the EASI score at 3 months and 6 months.	% Change in the EASI score at 3 months: −8.8% (20 mg apremilast BID). % Change in the EASI score at 6 months: −8.2% (30 mg apremilast BID) [237].	Completed.
			NCT02087943 (Phase II)	% Change in the EASI score at week 12.	% Change in the EASI score at week 12: −31.57% (apremilast 40 mg), −25.99% (apremilast 30 mg), −10.98% (placebo). Only 40 mg was significant vs. placebo [165].	Drug development program has been stopped.
			NCT00931242 (Phase II, no placebo arm)	IGA success at week 12.	20% of patients reached the primary endpoint (apremilast 20 mg) [238].	Completed.
			NCT03160248 (Phase II, nummular eczema)	PGA decrease of 2 or more points from baseline or absolute PGA 0/1.	Pending [239]	Completed
AA	CRTH2	QAW039/Fevipiprant	NCT01785602 (Phase IIa)	% Change in the EASI score at week 12.	EASI in treatment group declined 8.65%, compared to placebo group decline of 6.95% [240].	Drug development program has been stopped.
		OC000459	NCT02002208 (Phase IIb)	% Change in the EASI score at week 16.	EASI in treatment group declined 3.8%, compared to placebo group decline of 6.1% [241]	Drug development program has been stopped.
	COX and LOX	DS107/DGLA (dihomo gamma linolenic acid)	NCT02211417 (Phase II)	IGA score at week 8.	Pending.	Completed.
			NCT02864498 (Phase II)	IGA score at week 8.	Pending.	Completed.
			NCT03817190 (Phase II)	IGA success and achieving EASI75 at week 16.	A numerically higher proportion of patients in treatment group achieved the primary endpoint, but results were not statistically significant [242].	
Pruritus	IL-31RA	Nemolizumab	NCT01986933 (Phase III)	% Change in visual-analog scale (VAS) score for pruritis at week 12.	VAS% change:−42.8% in nemolizumab vs. −21.4% in placebo group [189]	Completed.
			NCT03985943 (Phase III)	IGA success and achieving EASI75 at week 16.	In progress.	In progress.
	IL-31	BMS-981164	NCT01614756 (Phase I)	Adverse event and % change in the EASI score from baseline to week 12.	Pending.	Completed.
	NK1R	VLY-686/tradipitant	NCT02004041 (Phase II)	% Change in the VAS score for pruritis at week 4.	Tradipitant was not superior to placebo in reducing itch intensity in patients with AD [243].	Completed.
			NCT02651714 (Phase II)	% Change in the VAS score for pruritus at week 8.	Primary endpoint: 44.2% in tradipitant vs. 30.6% in placebo; *p* = 0.019) [244].	Completed.
			NCT03568331 (EPIONE, Phase III)	Reduction in the I-NRS score at week 8.	No significant differences compared to placebo. Significant differences in participants with mild AD (IGA score of 1 or 2 at baseline) [194].	Completed.
			NCT04140695 (EPIONE2, Phase III)	Reduction in the WI-NRS score from baseline to week 8.	Pending.	Completed.
		Serlopitant	NCT02975206 (ATOMIK, Phase II)	Change in the WI-NRS score at week 6.	Change in the WI-NRS score was not significantly different between groups: −2.25 (serlopitant 5 mg), −2.32 (serlopitant 1 mg), −2.01 (placebo) [245].	Completed.
	H4R	ZPL-3893787/ Adriforant	NCT02424253 (Phase II)	Change in the I-NRS score at week 8.	Change in I-NRS was not significantly different between groups: −3.03 (ZPL-3893787), −2.66 (placebo); but change in EASI was significant (−50% in ZPL-3893787 vs. −27% in placebo) [193].	Completed.
	OPRK1	Difelikefalin	NCT04018027 (Phase II)	Change in the I-NRS score at week 12.	Primary endpoint was met in subset of participants with mild AD (BSA < 10) [195].	Completed, phase III programmed.

The table summarizes the results of the most advanced clinical trials for each drug

*AA* arachidonic acid, *BSA* body surface area, *COX* cyclooxygenase, *CRTH2* prostaglandin D2 receptor 2, *EASI* Eczema Area and Severity Index, *EASI 75* ≥75% reduction in the EASI score from baseline, *IGA* Investigator Global Assessment, *IGA success* clear or almost clear (grade 0 or 1) and at least a two-grade improvement in the IGA score from baseline, *I-NRS* Itch Numerical Rating Scale, *LOX* lipoxygenase, *PDE* phosphodiesterase, *PG* prostaglandin, *PGA* physician’s global assessment, *VAS* visual analog scale, *WI-NRS* Worst Itch Numeric Rating Scale

**Table 5:** Topical treatments for AD targeting the JAK-STAT axis and PDE

Axis	Target	Drug	Clinical trial (NCT number)	Primary endpoint	Results	Status of investigation
JAK-STAT	JAK 1/2/3, TYK2	Delgocitinib ointment	NCT03725722 (Phase II)	% Change in the EASI score at week 8.	EASI improvement was −1.9 vehicle group vs. −5.0 delgocitinib 1 mg/g vs. −4.9 delgocitinib 3 mg/g vs. −5.8 delgocitinib 8 mg/g vs. −7.6 delgocitinib 20 mg/g [246].	Completed.
			JapicCTI-173554 (Phase III)	% Change in the EASI score at week 24.	EASI change was −44.3% delgocitinib vs. 1.7% placebo [247].	Approved in Japan.
	JAK 1/2	Ruxolitinib cream	NCT03745638 (TRuE AD1, Phase III), adolescents and adults.	IGA success at week 8.	IGA success was achieved in 15.1% vehicle vs. 50% ruxolitinib 0.75% vs. 53.8% ruxolitinib 1.5% [152].	FDA approved for the treatment of mild-to-moderate AD in patients aged 12 years and older (September 2021)
			NCT03745651 (TRuE AD2, Phase III, and open-label long-term extension) adolescents and adults.	IGA success at week 8.	IGA success was achieved in 7.6% vehicle vs. 39% ruxolitinib 0.75% vs. 51.3% ruxolitinib 1.5% [152].	
	JAK 1/3	ATI-1777 solution	NCT04598269 (Phase II)	% Change in the EASI score at Day 28.	Pending.	Completed.
		Tofacitinib ointment	NCT02001181 (Phase IIa)	% Change in the EASI score at week 4.	EASI change was −81.7% tofacitinib vs. −29.9% vehicle [153].	Completed.
		Ifidancitinib (ATI-502) solution	NCT03585296 (Phase II), no vehicle group.	Safety and tolerability at week 8.	0% mortality, 4.55% serious adverse event (cellulitis, *n* = 1/22), 31.82% other TEAE (*N* = 7/22) [154].	Completed.
	JAK3, TrkA	SNA-125	Phase I/II	Safety and tolerability after 14 days.	No serious adverse events reported [248].	Phase II study was planned for 2019.
PDE inhibitors	PDE4	Crisaborole 2% ointment	NCT02118766 (AD-301, Phase III) participants 2 years and older.	IGA success at Day 29 and safety.	IGA success: 32.8% crisaborole 2% vs. 25.4% vehicle [167].	FDA approved for mild-to-moderate AD in participants as young as 3 months of age (March 2020).
			NCT02118792 (AD-302, Phase III) participants 2 years and older.	IGA success at Day 29 and safety.	IGA success: 31.4% crisaborole 2% vs. 18% vehicle [167].	
			NCT03645057 (Phase III) children.	Itch and pain at week 12.	Pending.	
			NCT04360187 (Phase III); Chinese and Japanese 2 years and older.	% Change in the EASI score at Day 29 and safety.	EASI change was −59.92% crisaborole 2% vs. −42.79% vehicle [249].	
			NCT04040192 (Phase III) children and adults.	Flare free maintenance and safety (week 52)	Pending.	
			NCT04498403 (Phase III) Japanese children and adults.	TEAEs at week 12.	TEAEs: 30.0% crisaborole 2% (adults), vs. 36.7% crisaborole 2% (pediatric) [250].	
		Difamilast/ OPA-15406 ointment	NCT03908970 (Phase III)	IGA success at week 4.	IGA success: 38.46% difamilast 1% vs. 12.64% vehicle [251].	Completed.
			NCT03911401 (Phase III), pediatric.	IGA success at week 4.	IGA success: 44.58% difamilast 0.3% vs. 47.06% difamilast 1% vs. 18.07% vehicle [252].	Completed.
			NCT03961529 (Phase III) children and adults.	Safety (number of TEAEs up to week 52).	TEAEs: 72.3% difamilast 1% (adults), vs. 91.0% difamilast 0.3% (pediatric) vs. 83.9% difamilast 1% (pediatric) [253].	Completed.
		Roflumilast/ ARQ-151 cream	NCT01856764 (Phase IIa)	Change in SCORAD at Day 15.	Change in SCORAD was −2.30 roflumilast 0.5% vs. −1.75 vehicle (nonsignificant change), but significant difference was seen in pruritus symptom [254].	Completed.
			NCT00746382 (Phase II)	Improvement in clinical signs and symptoms score, improvement in pruritus severity.	Study withdrawn due to business decisions. No participants were treated.	No participants were treated.
		Lotamilast/ RVT-501/ E6005 ointment	NCT03394677 (Phase II) children	IGA, EASI, I-NRS, and BSA scores at Day 28.	Pending.	Completed.
			NCT02950922 (Phase II) adolescents and adults.	Safety and tolerance at Day 28.	Pending.	Completed.
			NCT01461941 (Phase II)	Change in the EASI score and pruritus at week 12.	Changes in EASI and pruritus were numerically higher in the treatment group compared to placebo group, but not statistically signifcant [255].	Completed.
		DRM02 gel	NCT01993420 (Phase II)	Change in physicians' lesion assessment at week 6.	Pending.	Completed.

The table summarizes the results of the most advanced clinical trials for each drug

*BSA* body surface area, *EASI* Eczema Area and Severity Index, *IGA* Investigator Global Assessment, *IGA success* clear or almost clear (grade 0 or 1) and at least a two-grade improvement in the IGA score from baseline, *I-NRS* Itch Numerical Rating Scale, *PDE* phosphodiesterase, *SCORAD* SCORing Atopic Dermatitis, *TEAEs* treatment-associated adverse events

**Table 6:** Topical treatments for AD targeting multiple axes

Axis	Target	Drug	Clinical trial (NCT number)	Primary endpoint	Results	Status of investigation
AA	PLA2 inhibitors	MRX-6 cream.	NCT02031445 (Phase II)	IGA score at week 4.	Interim analysis showed lack of efficacy.	Terminated.
		ZPL-5212372 ointment	NCT02795832 (Phase II)	% Change in the EASI score up to Day 15.	No difference in %EASI compared to vehicle (only if worst case imputation analysis) [256]	Completed.
	LT	Q301/zileuton cream	NCT02426359 (Phase II) adolescents and adults.	% with an IGA score of 0/1 at week 8.	Pending.	Completed.
			NCT03571620 (Phase II)	% with an IGA score of 0/1 at week 8.	Pending.	Completed.
	COX and LOX enzymes	DS107/DGLA (dihomo gamma linolenic acid, diroleuton) cream	NCT02925793 (Phase II)	Change in the NRS-pruritus and EASI scores at week 8.	Pending.	Completed.
			NCT03676036 (Phase II)	EASI score at week 5.	Pending.	Completed.
			NCT03676933 (Phase II)	IGA score and SCORAD at week 9.	Pending.	Completed.
AhR	AhR	GSK2894512/ WBI-1001/ DB06083/ Tapinarof cream	NCT02564055 (Phase II)	IGA success at week 12.	Primary endpoint was statistically significant for tapinarof 1% BID (58% vs. 24% vehicle BID) [173].	Completed.
			NCT01098734 (Phase II)	IGA score of 0/1 at week 6.	Pending.	Completed.
			NCT05326672 (Phase III)	IGA success at week 8.	Pending	In progress.
T-cell	T cells activity	SP14019, topical cyclosporine 5% solution	NCT02865356 (Phase II)	Change in EASI, IGA and ADSI scores at Day 7, 14, 21, 28.	Pending.	Completed.
Antibacterial activity	AMP	Omiganan gel/CLS001 (indolicidin analog)	NCT02456480 (Phase II)	Pharmacodynamics, VAS-pruritus score, SCORAD, change in lesion size.	SCORAD change: −18.5% omiganan gel vs. −8.5% vehicle. Significant changed were noted in VAS-pruritus, skin microbiota and other clinical scores [257].	Completed.
			NCT03091426 (Phase II)	SCORAD, EASI, IGA, POEM, and DLQI scores, eDiary, Clinical Photography, and pharmacodynamics at 7 weeks.	A reduction in cultured *S. aureus* was observed in all omiganan treatment groups, with a significant reduction for omiganan 2.5%. No significant clinical improvement was observed [258].	Completed.
Pruritus	TrkA	CT327/ SNA-120/ pegcantratinib	NCT01808157 (Phase II) ointment.	VAS-pruritus score at week 4.	Pending.	Completed.
			NCT00996008 (Phase II) cream.	% Change in the EASI score at Day 14.	Terminated: due to slow recruitment, study was stopped before target enrollment was achieved.	Terminated.
	TRPV1	Asivatrep/ PAC-14028 cream	NCT02583022 (Phase II)	SCORAD change at Day 28.	Pending.	Completed.
			NCT02757729 (Phase II)	IGA score of 0/1 at week 8.	IGA 0/1 success rates were 14.58% vehicle cream vs. 42.55% PAC‐14028 cream 0.1% vs. 38.30% PAC‐14028 cream 0.3% vs. 57.45% PAC‐14028 cream 1.0% [208].	Completed.
			NCT02965118 (CAPTAIN-AD, phase III) adolescents and adults.	IGA score of 0/1 at week 8.	Pending.	Completed.
		1.5% PEA cream	NCT05003453 (phase II, III)	EASI change at week 2 and week 4.	Pending.	Completed.
Other mechanisms	IPCs analog	DMT210 gel	NCT02949960 (Phase II)	ADSI of each Target lesions at Day 28.	Pending.	Completed.
	Liver X receptor agonist	VTP-38543 cream	NCT02655679 (Phase II)	TEAEs and tolerability at Day 28.	TEAEs: 5% VTP-38543 0.05% vs. 15.8% VTP-38543 0.15% vs. 10.0% vehicle with transcutol vs. 25% VTP-38543 1% vs. 10.0% Vehicle without transcutol) [259]. Improvement in barrier differentiation and lipids was noted.	Completed.
	Serotonin B2 receptor antagonist	AM1030 cream	NCT02379910 (Phase II)	TEAEs and tolerability at Day 15.	Pending.	Completed.

The table summarizes the results of the most advanced clinical trials for each drug

*AA* arachidonic acid, *ADSI* Atopic Dermatitis Severity Index, *AhR* aryl hydrocarbon receptors, *AMP* antimicrobial peptide, *COX* cyclooxygenase, *EASI* Eczema Area and Severity Index, *IGA* Investigator Global Assessment, *IGA success* clear or almost clear (grade 0 or 1) and at least a two-grade improvement in the IGA score from baseline, *I-NRS* Itch Numerical Rating Scale, *PEA* palmitoylethanolamide, *PG* prostaglandin, *IPCs* isoprenylcysteines, *LOX* lipoxygenase, *LT* leukotriene, *PLA2* phospholipase A2, *SCORAD* SCORing Atopic Dermatitis, *TEAEs* treatment-associated adverse events, *TrkA* tropomyosin receptor kinase A, *TRPV1* transient receptor potential cation channel subfamily V member 1, *VAS* visual analog scale

### The Th2 pathway

The immune activation of the Th2 pathway is the hallmark of AD: a strong Th2 tone is the basis of all AD phenotypes and endotypes. The Th2 response is triggered by environmental irritants and allergens that penetrate the skin barrier. Dendritic and Langerhans cells in the skin sense these environmental factors and release cytokines that activate Th2 cells. Th2 cell activation is associated with an upregulation of the cytokines IL-4, IL-13, IL-5, IL-31, IL-33, OX40 and OX40 ligand (OX40L). Treatments targeting the Th2 pathway are summarized in Table 1. IL-33 will be discussed among the IL-1a family members, and IL-31 will be discussed among the pruritus-related targets.

IL-4 and IL-13 are considered the main drivers of the Th2 immune axis and key cytokines for the pathogenesis of AD. In murine models, they induce an AD-like phenotype in the epidermis characterized by pruritus, xerosis, inflammation, and increased *Staphylococcus aureus* infections [64–66]. These cytokines are upregulated in lesional and nonlesional AD skin, and their expression correlates with disease severity [67]. In AD skin, IL-4 and IL-13 contribute to skin barrier impairment, reducing the expression of terminal differentiation proteins such as FLG, LOR, and involucrin (INV) in both lesional and nonlesional skin [68, 69]. They act on B cells, inducing IgE class switching and production and sustaining Th2 activation [70]. IL-13 also stimulates dermal fibroblasts to produce collagen, contributing to AD-related skin fibrosis [71].

#### IL-4Rα

IL-4Rα mediates the signaling of both IL-4 and IL-13 and thus represents an ideal target. Dupilumab is an IgG4 monoclonal antibody that binds to IL-4Rα and is currently approved by the FDA for the treatment of AD in patients 6 months and older. The most important phase III trials and corresponding results that led to dupilumab approval are summarized in Table 1. In all these trials, conjunctivitis was the most frequent side effect [54–56].

Another IL-4Rα inhibitor known as CBP-201 is currently being studied in two phase II trials (NCT04444752, NCT05017480), and promising results have been released [72]. CBP-201 showed superiority to placebo in inducing a mean change in EASI scores of −63.0% in the CBP-201 every 2 weeks (Q2W) group and −65.4% in the CBP-201 every 4 weeks (Q4W) group vs. −40.7% in the placebo group [72]. CM310 is another anti-IL-4Rα monoclonal antibody currently under phase II (NCT04805411) and phase III study (NCT05265923, NCT04893707).

#### IL-13

Tralokinumab is an IgG4k IL-13 inhibitor that blocks IL-13 by preventing it from binding IL-13Rα1 and IL-13Rα2 [73]. It was approved by the FDA in December 2021 for the treatment of moderate-to-severe AD in adult patients. Six phase III clinical trials have been completed (see Table 1), and three more are still active. Upper respiratory tract infections and conjunctivitis were the most commonly reported adverse events [74].

Similarly, lebrikizumab is an IgG4k monoclonal antibody that binds specifically to IL-13, preventing IL-13Rα1/IL-4Rα heterodimerization and consequent signaling [75]. Safety and efficacy in AD have been under investigation in a total of ten phase III trials (see Table 1). Recently, the results of two phase III trials, ADvocate1 and Advocate2, investigating lebrikizumab as monotherapy for moderate-to-severe disease have been presented [76]. Rates of IGA success (defined as an IGA score of 0 or 1 with at least a 2-point improvement compared to baseline) at week 16 were 43 and 33% in the lebrikizumab arm vs. 13 and 11% in the placebo arm in the two studies, respectively. The rates of achieving EASI75 were 59 and 51% in the lebrikizumab arm and 16 and 18% in the placebo arm in the two studies, respectively. Conjunctivitis and dry eye symptoms were the most commonly reported adverse events [77].

Cendakimab (anti-IL-13) and Eblasakimab (anti-IL-13Rα1) are currently in phase II of development, but the results are not yet available.

#### IL-5

IL-5 induces the migration of eosinophils, which have an important role in allergic diseases such as asthma and eosinophilic esophagitis. However, the pathogenic role of eosinophils in AD is still unclear [78]. The inflammatory infiltrate of AD shows the presence of eosinophils, and skin eosinophil numbers are particularly increased in patients with an onset of AD before adulthood [79, 80]. A high number of AD patients show elevated eosinophil levels in the blood, which appear to correlate with disease severity [81]. Eosinophil blood levels are more pronounced in patients with extrinsic AD and associated respiratory allergic disease [46, 82, 83].

Mepolizumab is an IgG1k IL-5 inhibitor that was studied in a phase II clinical trial, which has since been terminated for reaching the futility criteria: mepolizumab did not reach primary endpoints of clinical improvements after 16 weeks of treatment, despite achieving a significant decrease in the number of peripheral blood eosinophils (NCT03055195; Table 1) [84]. The decrease in the number of blood eosinophils may have a negligible impact on resident skin eosinophils, or their activation status or mepolizumab concentration in the skin might not have been optimal [84]. Another possible explanation is that mepolizumab may be more effective in preventing flares in stabilized AD than in controlling active disease, as was the case in asthma patients [84].

Similarly, benralizumab interferes with IL-5 activity, blocking IL-5Rα. Benralizumab has been investigated in two phase II trials, one of which has been completed; however, the results have not yet been released. (NCT03563066, NCT04605094).

#### TSLP

TSLP is a cytokine produced in response to proinflammatory stimuli that potentiates Th2 skewing. It is overexpressed by the keratinocytes of patients with acute or chronic AD and acts on many immune cells (mast cells, DCs, and natural killer cells), inducing the production of IL-4, IL-5, IL-13 and TNF-α [85–87]. TSLP also induces DCs to express OX40L, which binds to OX40 on T cells to further stimulate the production of Th2-related cytokines [86, 88].

Tezepelumab (MEDI9929/AMG157) is a monoclonal antibody that targets TSLP. It was investigated in a phase IIa trial in combination with TCSs for 12 weeks. After 12 weeks of treatment, a higher number of patients in the treatment arm (64.7% vs. 48.2% in the placebo arm) reached the primary endpoint of achieving EASI50 at week 12, but the results failed to reach statistical significance [88]. The most common adverse event in the treatment group was nasopharyngitis (NCT03809663) [88]. Since preclinical studies implicate a role for TSLP in AD, the limited efficacy seen in this study is surprising. Several factors might have contributed to this outcome; for instance, only 20% of the enrolled patients had a severe form of the disease [88]. It is also possible that the concomitant use of TCSs (which was higher in the placebo-treated patients) might have caused a higher placebo effect [88]. Finally, given the upstream mechanism of action of TSLP, a longer treatment period may reveal greater improvements in AD symptoms [88].

#### OX40/OX40L

OX40 belongs to the tumor necrosis factor (TNF) receptor superfamily and acts as a costimulatory receptor. It is expressed upon activation by T cells, including effector T cells and regulatory T cells (Tregs). In inflammatory states, OX40L is expressed by TSLP-activated antigen-presenting cells, including DCs and endothelial cells [14, 89–91]. The OX40–OX40L interaction induces the expansion and prolonged survival of effector T cells, suppressing their apoptosis, promoting their activation, and inducing cytokine production, including Th2 cytokines (IL-4, IL-5, IL-13, and IL-31) [90, 92]. It also promotes and sustains the expansion of Th2 central memory cells and facilitates T-cell adhesion and migration [93]. Blocking this receptor‒ligand interaction prevents the subsequent activation of the Th22 pathway following Th2 activation and may also encourage T-cell tolerance and regulatory T-cell (Treg) proliferation [92].

GBR830 is an anti-OX40 monoclonal IgG1 antibody that has undergone phase IIa testing with promising results. In this study, patients were randomized to receive two doses of placebo or GBR830 28 days apart. In the treatment group, the rates of achieving EASI50 at Day 71 were significantly higher than those in the placebo group (76.9% vs. 37.5%). The GBR830 group was found to have a treatment-emergent adverse event (TEAE) distribution that was similar to that of the placebo group [94].

Amlitelimab (KY1005) is an anti-OX40L monoclonal antibody currently undergoing phase II testing (NCT03754309, NCT05131477). The results of a phase IIa clinical trial (NCT03754309) showed that the drug was superior to placebo: the average improvement in the EASI score from baseline was 80% for the amlitelimab-low dose treatment group and 70% for the amlitelimab-high dose group vs. 49% in the placebo group after 12 weeks of treatment [95]. Moreover, amlitelimab decreased IL-22 serum levels over the course of the study, but IL-22 baseline serum levels did not differ between responders and nonresponders [96].

Rocatinlimab (KHK4083/AMG451) is an anti-OX40 monoclonal antibody that showed promising results in a 16-week-long phase II trial (NCT03703102) [97]. Improvement in the EASI score from baseline (%) and rates of achieving EASI75 and IGA success were higher in the KHK4083 group than in the placebo group at the end of the treatment period. Interestingly, additional improvement was observed after week 16. Pyrexia, chills, nasopharyngitis, and AD worsening were the most frequent adverse events, similar to those reported in the phase I trial [98]. On a molecular level, rocatinlimab was demonstrated to reduce not only Th2/Th22-related markers in serum (CCL17, IL-22) but also pruritus-related molecules (neurturin, neurotrophin-3). In addition, skin transcriptomic analysis revealed a reduction in the expression of OX40 and Th2- (IL-13, IL-31, CCL17) and Th1/Th17/Th22-related markers (the Th1-associated transcription factor Tbet, IL-17A, and IL-22) and was demonstrated to improve the expression of skin barrier molecules (FLG, CLD23) [99].

### The Th17/IL23 pathway

The Th17/IL23 axis is the primary target for the treatment of psoriasis; however, it is also upregulated in AD. Several AD phenotypes, including intrinsic AD, Asian AD, and pediatric AD phenotypes, exhibit a higher Th17/IL23 skew [28]. AD lesional skin shows an upregulation of cytokines and molecules belonging to the Th17 axis (such as IL-17A, IL-12/23p40 and IL23p19, CCL20, PI3/Elafin, lipocalin-2) compared to the skin of healthy controls [25, 100]. IL-23 activates Th17 cells, which regulate AMP production by keratinocytes and induce the downstream production of IL-17 and IL-22 [25, 94, 100]. IL-17 and IL-22 worsen AD by downregulating FLG and other genes important for cellular adhesion, which increase skin barrier dysfunction and contribute to tissue inflammation [101]. Treatments targeting the Th17/IL23 pathway are summarized in Table 2.

#### IL-23

Ustekinumab (anti-IL-12/23p40) and risankizumab (anti-IL-23p19) are currently approved for the treatment of plaque psoriasis. Their efficacy in the treatment of AD has been investigated in phase II clinical trials (NCT01945086 [102], NCT01806662 [103] for ustekinumab and NCT03706040 [104] for risankizumab); however, they failed to reach the primary endpoints [105]. Ustekinumab was not superior to placebo in meeting the primary endpoints (achieving SCORAD 50 [103] and a change in the EASI score from baseline [102]) [105]. However, a transcriptomic investigation showed significant modulation of AD-related immune pathways (Th2, Th17, Th22, and Th1) with robust immune and inflammatory gene downregulation and improvement in terminal differentiation and skin hyperplasia after ustekinumab treatment [103]. Possible reasons for the lack of clinical efficacy may be that ustekinumab, used at the dosages recommended for psoriasis, might be underdosed for AD, and the use of TCSs might have contributed to a high placebo effect [103]. The results of a phase II clinical trial investigating the efficacy of risankizumab in the treatment of AD (NCT03706040) also showed no differences in the primary endpoint (achieving EASI75) between the placebo and risankizumab groups [104].

#### IL-17A

Secukinumab, an anti-IL-17A antibody currently used for the treatment of plaque psoriasis, has been investigated in the treatment of AD in a pilot study (NCT02594098) [106]. Ultimately, the results of this study demonstrated no significant differences between the secukinumab group and the placebo group in clinical improvements (changes in SCORAD and the EASI score from baseline) at week 16 [106]. In line with this finding, no significant differences were noted in epidermal thickness, epidermal hyperplasia (K16, ki67), or immune cell infiltration between the secukinumab and placebo groups. The AD skin transcriptomic profile was minimally impacted by secukinumab treatment, and no significant differences were observed in the extrinsic vs. intrinsic AD groups or in the Asian patient subgroup. Based on these results, targeting IL-17 alone does not seem to be sufficient to successfully treat AD. Another phase II trial has been completed, but results have yet to be published (NCT03568136).

### The Th22 pathway

The Th22 axis is directly involved in AD pathogenesis: both Th22 cells and Tc22 cells have a pathogenetic role and are present in increased numbers in skin samples from patients with AD compared to healthy controls [107]. Both acute and chronic AD lesional skin express higher levels of IL-22 [107–109]. The activation of the Th22 pathway is believed to play a key role in linking the barrier and the immune defects in AD. Th22 cells produce IL-22, which contributes to skin barrier damage by acting on keratinocytes, inhibiting their differentiation and promoting epidermal hyperplasia [100, 107–110]. IL-22 skin expression correlates with disease severity and response to treatment [107–109]. Treatments targeting the Th22 pathway are summarized in Table 2.

#### IL-22

Fezakinumab is an anti-IL-22 monoclonal antibody that was tested in a phase IIa trial [111]. The primary endpoint, measured as the change in SCORAD from baseline, was not met after 12 weeks of treatment; however, it reached statistical significance at week 20, 10 weeks after the last fezakinumab dose [111]. Upper respiratory tract infections were the most frequently reported adverse event [111]. When patients were analyzed according to severity at baseline, the patients with severe AD showed significant SCORAD improvement compared to the placebo group as early as week 6 [111]. Moreover, IL-22 blockade with fezakinumab was demonstrated to induce a reversal of the AD genomic profile [112]. When participants were stratified based on baseline skin IL-22 mRNA expression, high baseline IL-22 patients showed a stronger molecular response [112]. In this subset of patients, there was a significant downregulation of multiple AD-associated immune axes (Th2, Th22, Th17, and Th1), similar to what was observed with dupilumab treatment. Overall, these results represent an example of the application of a personalized medicine approach and confirm IL-22 as a pathogenetic cytokine in AD. These observations highlight how a clear definition of assessment time points and patient stratification are important to determine treatment efficacy.

#### IL-17C

The IL-17 family consists of a total of 6 members (IL-17A-F, with IL-17E also called IL-25) [113]. IL-17C is produced mainly by epidermal keratinocytes, while IL-17A is produced mainly by Th17 lymphocytes [114]. The effects of IL-17A and IL-17C on keratinocytes are similar: they induce the production of S100A proteins, AMPs, CXCL1, CCL20, IL-36 IL-1, and IL-8 and stimulate epidermal hyperplasia [41, 115]. IL-17C further potentiates Th17 activation in an autocrine and paracrine manner, favoring the production of IL-17A, IL-17F, and IL-22. IL-17C and IL-17A mutually influence one another’s production: IL-17C induces IL-17A synthesis in T lymphocytes, and IL-17A induces IL-17C synthesis in keratinocytes [114].

MOR106 is an anti-IL-17C monoclonal antibody that demonstrated promising results in a phase I study and a favorable safety profile [116]. However, the phase II clinical trial (NCT03864627) was terminated due to a low probability of meeting the primary endpoint. All clinical developments of MOR106 have now been discontinued [117].

### The IL-1 family

IL-1 is a proinflammatory cytokine that plays a central role in innate immunity [118]. The IL-1 family includes 11 cytokines, IL-1α, IL-1β, IL-1Rα (IL-1 receptor antagonist alpha), IL-18, IL-33, IL-33, IL-36α, IL-36β, IL-36γ, IL-36ra, IL-37, and IL-38 [118]. The receptor for IL-1α and IL-1β is IL-1R1, that for IL-33 is ST2 (also called interleukin-1 receptor-like 1, IL-1RL1), and that for IL-36 is IL36R (also called interleukin-1 receptor-like 2, IL1-RL2). IL-1Rα exerts an anti-inflammatory effect due to its ability to bind to IL-1R1, preventing IL-1α and IL-1β signaling [118]. IL-1α and IL-1β are produced by cells belonging to the innate immune system, including macrophages and monocytes [119]. IL-1 is abundantly expressed in the skin, and the balance between IL-1Rα and IL-1 is important in the maintenance of epidermal homeostasis [120]. Inflammatory cutaneous diseases, including psoriasis, alopecia areata, and AD, are associated with increased IL-1 expression in the skin [121, 122]. Exposure to external agents such as house mouse allergens or UVB has been shown to induce increased IL-1 secretion from keratinocytes [123]. IL-1 contributes to Th17 and Th2 cell development and to the chronification of AD lesions through an increase in Th1 shifting [118, 124].

The IL-1 family can be divided into three different subfamilies: the IL-1, IL-18, and IL-36 subgroups. The IL-36 subgroup is composed of IL-36α, IL-36β, IL-36γ, IL-36RN (alias IL-36RA), and IL-38. AD lesional skin shows higher levels of IL-36 and IL-36RN [125]. IL-36 cytokines are produced mainly by keratinocytes, and they sustain and propagate skin inflammation: they induce the production of chemokines that drive immune cell chemotaxis, including macrophage and T-cell chemokines (CCL2, CCL3, CCL4, CCL5, CCL17, CCL22, CCL20) and neutrophil chemokines (IL-8, CCL20, and CXCL1); activate and induce antigen-presenting cells to produce IL-1 and IL-6; and indirectly drive T-cell proliferation [126]. Treatments targeting the IL-1 family are summarized in Table 2.

#### IL-1α

Bermekimab is an anti-IL-1α monoclonal antibody that has been investigated in a phase II, open-label, dose-escalation study (NCT03496974) [127]. In this study, two active treatment cohorts, bermekimab 200 mg and bermekimab 400 mg, were compared. The primary endpoint was safety, measured as the number of patients exhibiting TEAEs. Thirty percent of patients in the cohort receiving 200 mg of the drug compared to 21.4% of patients receiving 400 mg of the drug had TEAEs. Improvements in the mean change in EASI scores from baseline and other severity scores (IGA, pain, and pruritus scores) were reported with a higher magnitude for the 400 mg group than the 200 mg group [127]. Another phase II trial comparing placebo, bermekimab 200 mg every week and bermekimab 200 mg every other week is currently in progress (NCT04021862).

#### IL-36

Spesolimab (BI 655130) is a humanized monoclonal IgG1 that binds to IL-36R. Two phase II clinical trials investigating spesolimab for the treatment of AD (NCT03822832, NCT04086121) have been completed. One of these studies (NCT04086121), enrolling a total of 14 patients, evaluated the long-term safety of spesolimab: TEAEs were reported in 64.3% of patients, with nasopharyngitis being the most common. EASI50 was reached by 33.3% of patients [128].

#### IL-33

IL-33 is released by keratinocytes and contributes to both inflammation and skin barrier disruption in AD. Exposure to allergens or staphylococcal toxins induces high levels of IL-33 in AD skin [129–131]. IL-33 downregulates FLG expression; favors the activation of Th2 lymphocytes; increases the production of IL-4, IL-5, and IL-13; amplifies the TSLP-OX40L axis; and activates mast cells and eosinophils, ultimately potentiating Th2 “responses [129–131].

Etokimab, a G1k humanized anti-IL-33 monoclonal antibody, was evaluated in a 16-week-long, phase IIb clinical trial but failed to achieve its primary endpoint and is no longer being tested for the treatment of AD (NCT03533751) [132]. Another IL-33 inhibitor, itepekimab (REGR3500), failed to reach significance in a phase II clinical trial that was terminated owing to a lack of efficacy (NCT03736967) [133], while the results of another phase II monotherapy trial (NCT03738423) have been submitted and are awaiting quality control before publication. The inhibition of IL-33 has not been demonstrated to be able to impact the course of AD to date, while it has demonstrated some efficacy for the treatment of asthma, a common AD comorbidity [134].

### The JAK-STAT signaling pathway and SYK

Janus kinases (JAKs) and signal transducer and activator of transcription proteins (STATs) modulate the intracellular signaling of key cytokines implicated in AD, such as Th2 (IL-5, IL-4, IL-13), Th17 (IL-17A, IL-17F, IL-21), Th22 (IL-22) and Th1 (IFN-γ, IL-2, TNF-β) cytokines [135]. Four kinases, JAK1, JAK2, JAK3, and TYK2 (tyrosine kinase 2), belong to the JAK-STAT family. After the phosphorylation of their intracellular receptors, they mediate the activation of transcription factors belonging to the STAT family (STAT1, STAT2, STAT3, STAT5A/B, STAT6) and their translocation to the nucleus.

JAK1 and JAK3 mediate the signaling of IL-2, IL-4, IL-7, IL-9, IL-15 and IL-21 [136]. Intracellular signaling activated by IL-4 is mediated by a complex interaction of STAT3, STAT5, and STAT6 [137, 138]. STAT6 mediates responses that lead to Th2 and Th9 differentiation and IgE class switching on B cells [138]. Genetic variants of STAT6 have been linked to allergic diseases, including AD and increased IgE levels [139]. IL-13 signaling is mediated by JAK1/2 and TYK2, while IL-31 signaling is mediated by JAK1 and JAK2 [135]. JAK2 also mediates the signaling of IL-3, IL-5, and GM-CSF, while TYK2 mediates IL-23, IL-12, IFNs, and IL-6 signaling [139]. Th1 differentiation is mediated by JAK1, JAK2 and TYK2. IL-12, IL-23 and type 1 IFNs signal through the activation of STAT4 [139]. Overall, STATs are also important for mediating the signals of innate immunity, Th17 differentiation, regulatory T-cell differentiation, and CD8 T-cell and B-cell function [135]. JAK inhibition has been associated with a substantial antipruritic effect for which different mechanisms have been proposed. The stimulation of IL-4Rα expressed on sensory neurons and the subsequent activation of JAK1 may induce itch transmission [140]. JAK inhibition may also decrease itch transmission by modulating the signaling of TRPV1 (transient receptor potential vanilloid receptor 1) expressed on dorsal root ganglia (DRGs) [141]. Mouse models also support a role for JAK1 inhibition in controlling itch [140].

SYK, also known as spleen tyrosine kinase, is involved in Th17 signaling and in keratinocyte differentiation: it stimulates Th17 cell recruitment in the skin, inducing keratinocytes to produce CCL20 [142]; regulates epidermal growth factor receptor signaling; and negatively affects keratinocyte differentiation [143]. SYK is also involved in B-cell responses, along with dendritic cell differentiation [144, 145].

Given the broad effect of JAK and SYK on multiple immune pathways, therapies targeting this axis have been investigated for the treatment of a variety of inflammatory and autoimmune diseases, such as psoriasis, alopecia areata, and AD. Currently, one topical JAK-STAT inhibitor (ruxolitinib) for mild-to-moderate AD and two oral formulations (abrocitinib and upadacitinib) for moderate-to-severe AD are approved by the FDA. Complete lists of JAK-STAT inhibitors investigated for the treatment of AD, including oral small molecules and topical treatments, can be found in Tables 5 and 6, respectively.

Abrocitinib is a JAK1 inhibitor that, in January 2022, was approved by the FDA for adult patients, and it is currently undergoing phase III testing in comparison with dupilumab (NCT04345367, NCT03720470). The JADE COMPARE study (NCT03720470) was not designed to make direct comparisons between abrocitinib and dupilumab; consequently, direct conclusions on superiority or inferiority cannot be made [146]. However, abrocitinib demonstrated rapid efficacy in treating AD, especially in patients with difficult-to-treat locations of disease. Regarding safety, nausea, upper respiratory infections, and headache were the most commonly reported side effects [62, 147]. Abrocitinib induced a transient dose-dependent decrease in platelet counts that was not associated with bleeding or other clinically relevant events [62, 147].

In January 2022, upadacitinib, another JAK1 inhibitor, was approved for patients 12 years and older. In a 16-week, phase III trial of upadacitnib compared to dupilumab, EASI75 was evaluated as the primary endpoint [148]. EASI75 was achieved by significantly more upadacitinib-treated patients than by dupilumab-treated patients (71% vs. 61.1%, respectively). Upadacitinib demonstrated superiority to dupilumab in all secondary endpoints, including improvement in the Worst Itch Numeric Rating Scale (WI-NRS) score (31.4% vs. 8.8%) and the percentage of patients achieving EASI75 (43.7% vs. 17.4%) and EASI100 at week 16 (27.9% vs. 7.6%). The most commonly reported adverse events were acne, creatine phosphokinase (CPK) elevation, and upper respiratory tract infections [148].

Baricitinib is a JAK1/JAK2 inhibitor that is currently indicated for adults with moderate-to-severe AD in Europe and Japan. It has undergone different phase III trials (see Table 3). The BREEZE-AD5 trial (NCT03435081) [149] studied baricitinib monotherapy in patients with moderate-to-severe AD. The primary endpoint of achieving EASI75 was reached in 29.5% of patients receiving baricitinib 2 mg compared to 12.9% of the baricitinib 1 mg and 8.2% of the placebo groups. A higher number of adverse events, the most common being headache, increased blood CPK levels, and nasopharyngitis, occurred in the baricitinib-treated group [149]. Baricitinib is currently being tested in children and adolescent patients (BREEZE-AD-PEDS, NCT03952559).

Gusacitinib is an oral JAK/SYK inhibitor that showed superiority to placebo in a phase I clinical trial (NCT03139981) [150]. Treatment with gusacitinib induced a reduction in inflammatory serum markers belonging to the Th2 (IL‐13, CCL13, CCL17), Th17 (IL‐27, KYNU), and Th1 (CXCL9, CXCL10, CXCL11, IL12/23p40) axes [150]. The same axes were targeted in the skin, where gusacitinib reversed the AD transcriptome toward a nonlesional phenotype, reducing the expression of several inflammation markers [151]. Gusacitinib also improved skin thickness, skin hyperplasia (K16 expression) and T-cell (CD3) and dendritic cell (CD11c) infiltration [151]. The results of a phase II trial have been submitted but are not yet available (NCT03531957). Another phase II trial was terminated (NCT03654755).

Topical JAK inhibitors are also under investigation for the treatment of mild and moderate AD (Table 5). Ruxolitinib cream is a JAK1/JAK2 inhibitor that was approved by the FDA in 2021 in patients 12 years and older. It has undergone two phase III studies (TRuE AD1 and TRuE AD2, respectively NCT03745638 and NCT03745651) in which the primary endpoint was the percentage of participants achieving IGA success at 8 weeks. In TRuE AD1, IGA success was achieved at rates of 7.6%, 39%, and 51.3% for the placebo, ruxolitinib 0.75%, and ruxolitinib 1.5% groups, respectively. In TRuE AD2, IGA success was achieved at rates of 15.1%, 50%, and 53.8% in the same corresponding groups. The most common AE in the treatment groups was nasopharyngitis [152]. A phase III study in children (NCT04921969) is currently recruiting patients.

Tofacitinib is a JAK1/JAK3 inhibitor that has undergone phase II study in the form of an ointment formulation. At the end of a 4-week phase II trial, the primary endpoint of a percent improvement in the EASI score was evaluated: the EASI score in the treatment group declined by 81.7%, compared to 29.9% in the placebo group. Nasopharyngitis was the most common TEAE (NCT02001181) [153].

Ifidancitinib is a JAK1/JAK3 inhibitor that was tested in a phase II trial as a topical solution. Safety and tolerability were evaluated as the primary outcome at 8 weeks. The trial showed no mortality, a serious adverse event (cellulitis in one of the 22 patients), and other adverse events in 7 of the 22 patients (NCT03585296) [154].

Delgocitinib cream is a JAK1/JAK2/JAK3/TYK2 inhibitor currently in phase II testing. In the phase II trial NCT03725722, patients were randomized to receive vehicle cream or delgocitinib cream at different concentrations (1 mg/g, 3 mg/g, 8 mg/g, or 20 mg/g). EASI score improvement at week 8 in these respective groups was measured as −1.9, −5.0, −4.9, −5.8, and −7.6, demonstrating an encouraging dose‒response curve for delgocitinib. The incidence of adverse events was similar in the two groups. In Japan, topical delgocitinib is approved for AD, while several phase III trials of topical delgocitinib for the treatment of chronic hand eczema are currently recruiting (NCT05355818, NCT04871711, NCT04872101, NCT05259722, NCT0494984).

### IgE

In AD, the Th2-driven expression of cytokines IL-4 and IL-13 activates B cells to overproduce IgE antibodies [70]. External antigens and allergens absorbed into the skin come in contact with IgE attached to the surface of mast cells. This link causes the degranulation of mast cells and the release of histamine and other mediators that cause pruritus and sustain Th2 skewing [155–157]. In addition, CD8 T cells are activated by antigen-bound IgEs and are associated with increased disease length and severity [158]. Mast cells and DCs normally express the receptor for IgE, type I (FCεR1), but in AD skin, its expression is higher than in the skin of healthy individuals [159]. Patients with extrinsic AD show a positive correlation between SCORAD and IgE levels [26].

Drugs that target IgE represent a possible therapeutic approach for extrinsic AD (see Table 2). Omalizumab is a recombinant IgGk anti-IgE monoclonal antibody targeting the high-affinity receptor binding site on human IgE, and it has demonstrated variable and contrasting results in the treatment of AD. One meta-analysis, analyzing 15 studies, showed that only 43% of the patients met the clinical efficacy criteria (defined as achieving either SCORAD-50 or EASI75 or IGA success) after omalizumab treatment. Patients with lower IgE serum concentrations showed better responses [160]. Omalizumab was investigated for the treatment of severe AD in children in a phase IV study (NCT02300701). Omalizumab was found to induce a significant improvement in treated patients (measured as a decline in SCORAD at week 24) with a potent TCS-sparing effect compared to placebo [161].

Ligelizumab is a humanized IgG1k monoclonal antibody targeting the immunoglobulin constant epsilon region of the heavy chain of IgE (IGHE). Ligelizumab to treat the extrinsic endotype of AD is currently in phase II testing (NCT01552629).

### Phosphodiesterase 4 (PDE4)

PDE4 is involved in cyclic adenosine monophosphate (cAMP) catabolism in inflammatory and immune cells such as B and T lymphocytes, basophils, mast cells, eosinophils, monocytes, macrophages, neutrophils, and endothelial cells [162]. Low levels of cAMP inside these cell populations promote inflammation via AD-related pathways (Th1, Th2, Th17, and Th22) [163]. Thus, the inhibition of PDE4 leads to a persistent increase in cAMP levels and subsequently reduces T-cell activation and cytokine production, ultimately exerting an anti-inflammatory effect [164]. For these reasons, phosphodiesterase 4 (PDE4) is an important target of both topical and systemic treatment for AD (see Table 3 for systemic and Table 5 for topical PDE4 inhibitors).

Apremilast is a small molecule that has been studied in 4 phase IIa clinical trials for the treatment of AD and AD subtypes, such as nummular eczema (NCT02087943, NCT01393158, NCT00931242, NCT03160248), at different dosages. In a phase II trial (NCT02087943), 191 patients were randomized to three different treatment arms, placebo, apremilast 30 mg twice a day (BID) or apremilast 40 mg BID, and EASI score improvement after 12 weeks was 10.98%, 25.99%, and 31.57%, respectively [165]. Only the improvement registered in the apremilast 40 mg group was significant vs. placebo (NCT02087943); however, an independent safety monitoring committee discontinued apremilast 40 mg. Adverse events were more frequent in the apremilast 40 mg group, and the most commonly reported were nausea, diarrhea, headache, and nasopharyngitis [165]. A phase II study of apremilast in combination with dupilumab is currently recruiting (NCT04306965).

Crisaborole 2% ointment is a topical PDE4 inhibitor that is currently approved by the FDA for the treatment of AD in patients older than 3 months of age. Phase III testing showed significant IGA score improvement in the treatment group at 29 days (IGA score improvement of 32.8%) compared to the placebo group (IGA score improvement of 25.4%) [166, 167]. The most frequently reported adverse event was upper respiratory tract infection (NCT02118766) [167]. An intrapatient study demonstrated that crisaborole, but not vehicle, was able to act on the AD transcriptomic profile, decreasing the expression of Th2, Th17 and Th22 markers and reducing epidermal AD changes [166]. Several other PDE4 inhibitors are currently being tested (see Table 5).

### Aryl hydrocarbon receptor system

The aryl hydrocarbon receptor (AhR) system is a sensitive sensor abundantly and constitutively expressed in healthy skin. It regulates the skin’s response to environmental toxins, including dioxins and other exogenous and endogenous chemicals such as tryptophan photoproducts [168]. Based on the ligand and on the duration of the activation, AhR can exert antioxidative or oxidative activity [169]. For example, hazardous dioxins induce the translocation of cytoplasmic AhR into the nucleus, where it heterodimerizes with AHR-nuclear translocator (ARNT) and induces the transcription of CYP1A1, which degrades AhR ligands. However, dioxins are chemically stable and long-lived; therefore, CYP1A1 generates high amounts of reactive oxygen species (ROS) while trying to degrade them. On the other hand, some AhR ligands can activate nuclear factor-erythroid 2-related factor 2 (NRF2), which upregulates the expression of antioxidative enzymes (such as heme oxygenase 1, NADPH dehydrogenase, and quinone 1) that counteract ROS production. AHR/ARNT signaling also activates the OVO-like 1 (OVOL1) transcription factor and upregulates the expression of FLG, LOR, and INV [169].

In AD, IL-13 and IL-4 activate STAT6, which interferes with the translocation of the transcription factor OVOL1 and inhibits the OVOL1-induced upregulation of FLG, LOR, and INV. Some AhR agonists can inhibit IL-4/IL-13-mediated STAT6 activation and restore the expression of FLG, LOR, and INV [170, 171].

On immune cells, AhR is expressed in Th17 and Treg cells and upregulates the expression of IL-17A, IL-17F, and IL-22 [169]. AhR ligation affects Th17 and Treg cell differentiation, but outcomes are inconsistent in different experiments, most likely due to different effects depending on the dose and duration of AhR activation. In a mouse model, it was demonstrated that high doses of AhR agonists increased the production of IL-10 and FoxP3+ Tregs, while low doses did not induce Tregs but instead increased the percentage of IL-17-producing CD4 + cells [172].

It was suggested that different ligands and durations of activation are responsible for the duality of AhR activation in AD. While rapid-metabolizing AHR ligands that activate the AHR/ARNT/FLG axis may be beneficial in treating AD, other slow-metabolizing dioxins and environmental pollutants cause potent and long-lasting activation of the AHR axis, which exacerbates barrier dysfunction and aggravates AD, likely due to an abnormally accelerated keratinization process, epidermal acanthosis, the elongation of nerve fibers, and the production of pruritogenic artemin [169].

Tapinarof is a high-affinity AHR agonist that is currently being tested as a topical cream to treat AD in a phase II trial (see Table 6). Tapinarof exerts antioxidative activity via NRF2 activation and augments the expression of FLG and INV [169, 171]. In a 12-week trial (NCT02564055), patients receiving tapinarof 1% twice a day had a significantly greater improvements in IGA scores than those receiving placebo (58% vs. 24% vehicle) [173]. Folliculitis and upper respiratory tract infections were the most frequent adverse events [173]. A phase II trial in pediatric patients (NCT05186805) and 3 phase III clinical trials in children and adults with AD are currently recruiting patients (NCT05142774, NCT05014568, NCT05032859).

### Pruritis-related targets

Chronic pruritis is the most burdensome symptom of AD. The molecular mechanisms that lead to pruritus are very complex and, for some aspects, still unclear. A fundamental role is played by IL-31 production and IgE-activated mast cell degranulation of histamine [174]. Histamine triggers itch by stimulating unmyelinated C fibers [175]. The H4 histamine receptor is associated with the immunomodulation and chemotaxis of mast cells and eosinophils [176, 177]. When the H1 histamine receptor is activated, it stimulates TRPV1 and mediates the sensation of pruritus [178]. TRPV1 is expressed primarily in unmyelinated sensory neurons. The neuropeptide substance P is involved in the neurotransmission of itch when bound to its receptor, neurokinin 1 receptor (NK1R), which is expressed by keratinocytes, mast cells, endothelial cells, fibroblasts, and sensory nerve endings in the skin [179]. Phospholipase A2 and 12-lipoxygenase-dependent activation of TRPV1 on histamine-sensitive C fibers causes the release of substance P [179]. Opioid receptors are also involved in the modulation of itch. While μ-opioid receptors (MORs) are believed to transmit the sensation of pruritus, k-opioid receptors (KORs) are believed to mediate an antipruritic signal when activated [180, 181]. KORs are expressed not only in the peripheral nervous system but also in immune cells and in human skin [180–182]. Systemic and topical treatments targeting pruritus associated with AD are listed in Tables 4 and 6, respectively.

#### IL-31

IL-31 is considered the “itch cytokine”, as it plays a key role in the symptoms of pruritus in AD. In a mouse model, IL-31 was demonstrated to induce skin changes similar to those that occur in AD, characterized by epidermal hyperplasia, acanthosis, hyperkeratosis, and an increase in the number of inflammatory cells and mast cells [183]. IL-31 expression is increased in AD lesions and is thought to be responsible for perpetuating the itch-scratch cycle [184]. Mononuclear infiltrating cells were found to be IL-31-positive in samples from patients with severe AD [185]. IL-31RA was detected not only in the neurons of normal DRGs but also in dermal nerve fibers and in keratinocytes in the skin of AD patients [185]. IL-31 signals through a receptor complex composed of IL-31RA and OSMRβ. OSMRβ is also expressed in small nociceptive neurons in DRGs. Neurons that express OSMRβ also express TRPV1 and the transient receptor potential cation channel subfamily A, member 1 (TRPA1) [186–188]. IL-31RA is considered the key link between Th2-skewed inflammation and the neurotransmission of itch through the stimulation of sensory nerve endings [188].

Nemolizumab is an IgG2k antibody against IL-31RA that has undergone clinical trials in both adults and adolescents. In a phase III, double-blinded, randomized, placebo-controlled trial (NCT01986933), participants were randomized to receive nemolizumab or placebo in combination with TCSs as needed [189]. The primary endpoint was the change in visual analog scale (VAS) score for pruritus at the end of 16 weeks. The nemolizumab group had a significantly higher percent change than the placebo group (−42.8% vs. −21.4%) [189]. However, the change in the EASI score was nonsignificant, with a -45.9% change in the nemolizumab group compared to −33.2% in the placebo group. There was a slightly greater incidence of TEAEs with nemolizumab than with placebo (upper respiratory tract infections and gastroenteritis being the most common) [190]. No de novo cases of asthma were reported; however, there was a dose-dependent increase in asthma events in patients with a prior history of asthma. Another phase III trial is ongoing (NCT03985943) and will help clarify whether nemolizumab has a durable effect and is safe for AD.

#### OSMRβ

Oncostatin-M receptor β (OSMRβ) is the common receptor subunit for both IL-31 and oncostatin M (OSM) [108]. OMSRβ and gp130 are the two subunits of the type II oncostatin-M receptor (OSMR). OSMR is located on epidermal keratinocytes and is upregulated in skin lesions of both psoriasis and AD patients [108]. Its ligand, OSM, is mainly produced by dermal T cells and monocytes [191], and it induces the activation of intracellular signaling that involves the JAK1/2, TYK2, STAT1, STAT3, and MAPK pathways. It has a strong effect on keratinocytes that are activated to produce alarmins such as S100A proteins and beta-defensin 2, chemotactic proteins such as CXCL5 and IL-8 [108, 192] and products involved in tissue remodeling such as MMP1 and tenascin [192]. OSM upregulates the expression of IL-4, IL-4Rα, and other genes involved in Th2 inflammation and immunity [192]. It downregulates the expression of terminal differentiation genes (FLG, INV, LOR) and contributes to pruritus, enhancing neuronal excitability to pruritogens [191].

Vixarelimab (KPL-716) is a monoclonal antibody that targets OSMRβ. In a phase Ib study, vixarelimab demonstrated a rapid and sustained improvement in pruritus, measured as changes in WI-NRS scores; however, it was not able to induce significant changes in the AD-clinical scores (EASI or SCORAD) compared to placebo [190]. Vixarelimab is no longer being investigated for the treatment of AD but has been tested for pruritus in other dermatological conditions (NCT03858634). A phase II study is currently investigating vixarelimab for the treatment of pruritus in prurigo nodularis, but the results are not yet available (NCT03816891).

#### H4R

The H4 receptor inhibitor known as adriforant (ZPL-3893787) underwent phase II testing to treat itch associated with AD (see Table 4) [193]. Treatment with oral adriforant showed a higher reduction in EASI scores and a higher proportion of patients achieving IGA scores of 0/1 than placebo (50% vs. 27% and 18.5% vs. 9.1%, respectively) at week 8. Although adriforant demonstrated clinical efficacy in reducing AD severity scores, the 3-point reduction in pruritus with adriforant was similar to the reduction observed in the placebo group, resulting in a nonsignificant difference [193].

#### NK1R

Drugs that target NK1R attempt to block the neurotransmission of histaminergic itch signaling carried by substance P (see Table 4). Serlopitant is an oral NK1R antagonist that has undergone phase II testing to treat itch related to AD; however, the change in WI-NRS scores in the treatment group was not significantly different from that in the placebo group (NCT02975206). Similarly, tradipitant, another oral NK1R antagonist, was not superior to placebo in reducing itch intensity in AD patients in a phase III study (NCT03568331). However, a subanalysis demonstrated statistical significance for patients with mild AD, defined as an IGA score of 1 or 2 at baseline [194].

#### OPRK1

Difelikefalin (DFK) is an oral KOR agonist that was investigated for the treatment of moderate-to-severe pruritus in a phase II clinical trial (NCT04018027, see Table 4). A significant reduction in pruritus was observed in a subset of difelikefalin-treated patients characterized as having mild-to-moderate AD (BSA < 10%) [195]. Skin transcriptomic analysis showed a decrease in pruritus-related markers and in Th2-related markers in DFK-treated patients [196].

#### The cannabinoid receptor system

Cannabinoids exert an antipruritic effect through a combination of neuronal activation, the modulation of pruritus transmission pathways and local modulation of keratinocytes and mast cells. Cannabinoids can be classified into endocannabinoids (anandamide and 2-arachidonoylglycerol [2-AG]), phytocannabinoids (tetrahydrocannabinol, cannabidiol) and synthetic cannabinoids (such as palmitoylethanolamine).

Increased activity of the cannabinoid receptors CB1 and CB2 has been reported to alleviate pruritus. In contrast, the activation of TPRV1 increases pruritus [197]. The cannabinoid receptor CB1 mediates mainly an antipruritic effect in the central nervous system, while in the periphery, both CB1 and CB2 are thought to modulate analgesia and pruritus [197]. Topical cannabinoid agonists are thought to work both through peripheral nerve activity and by decreasing the recruitment of mast cells and subsequent histamine release [198, 199]. CB2 is widely expressed in immune cells, and its activation decreases inflammation [200]. CB1 agonism reduced Th-2-type cytokines in a mouse model of atopic dermatitis [201] and is believed to suppress mast cell activation, proliferation and degranulation [198, 202, 203]. No clinical trial of CB1/2 agonists for the treatment of AD is currently active.

TRPV1 is a member of the TRP receptor family and is the most studied TRP channel with regard to cannabinoid-mediated modulation of pruritus. Cannabinoids act as antagonists of TRPV1 and prevent neuronal activation by pruritic mediators [204]. Palmitoylethanolamine (PEA) is a ligand at TRPV1 channels with no direct interaction with CB1 and CB2 [205]. PEA and adelmidrol (a PEA analog) have been studied for the treatment of pruritus in AD in a large observational study [206] and in a small open-label study [207], respectively, with encouraging results. A phase II-III clinical trial evaluating the effectiveness of topical PEA for reducing atopic dermatitis severity compared to a base comparator moisturizer in adults has been completed (NCT05003453), but the results have not yet been released.

PAC-14028 cream is a selective TRPV1 antagonist that has undergone phase II testing for moderate-to-severe AD (see Table 6). Adult participants were randomized into four different treatment groups (vehicle cream, 0.1% PAC-14028 cream, 0.3% PAC-14028 cream, and 1% PAC-14028 cream), and IGA success rates at week 8 were 14.58%, 42.55%, 38.30%, and 57.45%, respectively. No significant safety issues were reported, and no clinically meaningful differences were found in the rate of the incidence of adverse events among the treatment groups (NCT02757729) [208]. The results of a phase III trial are pending (NCT02965118).

### Microbiota and bacterial therapy

Imbalances in the skin microbiome have been associated with skin barrier defects, namely, FLG gene mutations, and with increased inflammation in AD [209]. AD skin shows a loss of community bacterial diversity and a dominant proliferation of *S. aureus* [210]. Study findings suggest that early-life colonization with *S. aureus* predispose patients to the development of AD, while early-life commensal non-*S. aureus* colonization is protective [211]. *S. aureus* can penetrate the damaged AD skin barrier, and its proliferation results in increased inflammatory cytokines and the exacerbation of disease [18]. Disease severity correlates with greater skin colonization with *S. aureus* [212], and it has been demonstrated that anti-inflammatory therapy with dupilumab reduces the abundance of *S. aureus* [212]. Consistent application of skin emollients also affects the skin bacterial flora, inducing a restoration of a microbiome more similar to that of unaffected skin [213]. Therapies counteracting the proliferation of *S. aureus* have been promising in AD [214]; however, the clinical efficacy of antibiotics in this pathology remains questionable [215]. Other approaches are based on the use of prebiotics that can have a positive effect on the cutaneous microbiome [216]. In the past, the use of orally administered probiotics has been controversial [217], and other studies have addressed the utilization of bacterial lysates or heat-inactivated bacteria. More recently, studies have focused on the use of living bacteria or the topical use of certain strains of bacteriophages that can infect a wide spectrum of pathogenic *S. aureus* strains but do not attack *S. epidermidis* existing in symbiosis on human skin [218]. Another option is the use of bacteriophage endolysin (NCT02840955) or the combination of phages and surfactants [219].

Although the restoration of normal skin flora represents a well-known field in AD treatment [220], randomized controlled clinical trials are currently limited (see Table 6).

### Epigenetic modifications

“Epigenetic” is a term that comprises chromatin modifications such as DNA methylation, covalent modifications of histone proteins, and noncoding RNA-dependent actions that regulate the expression of genes within the genome. Epigenetic changes modulate the activation or inhibition of the transcription process of the genetic code without directly modifying the genetic code, and some studies suggest that epigenetic changes could be involved in non-Mendelian transgenerational inheritance [221, 222].

Changes in DNA methylation have been studied in keratinocytes and immune cells in AD [223]. Hypermethylation changes in the S100A proteins OAS2 and KRT6A were related to their increased expression in lesional skin. Other studies identified cell-specific methylation changes in CD4 + and CD8 + T cells in AD, some of which were able to influence the production of IL-13 and other inflammatory mediators [224, 225].

Histone acetylation has been studied in allergic diseases, especially allergic asthma, in which it influences the production of IL-13 and IL-8 [222]. More recently, butyric acid, a fermentation metabolite of *S. epidermidis*, was demonstrated not only to have an inhibitory growth effect on *S. aureus* but also to inhibit histone deacetylase (HDAC) activity, resulting in increased histone acetylation and upregulated gene expression [222].

Another active area of investigation is the role of miRNAs (microRNAs) that have been reported to modulate inflammatory factors in AD and, more recently, circular RNAs that indirectly regulate miRNA target genes and contribute to innate immune regulation [222].

To date, there are no data on therapies with drugs interfering with epigenetic processes in the treatment of atopic dermatitis. However, it is important to note that glucocorticosteroids, which are in extensive clinical use, can reduce the activity of HDAC, one of the key enzymes involved in epigenetic processes [226].

## Discussion

AD treatment is experiencing a translational revolution in which the link between bench research and clinical application has been fundamental. Not only the successful stories but also the negative results in clinical trials are useful to shape the direction of the research to better capture the biological variability of AD. There are some important considerations regarding the optimization of the study design of clinical trials for AD. First, given the upstream mechanism of action of some AD targets, longer time points might be needed to better capture the effect of certain molecules (e.g., fezakinumab). In addition, the concomitant application of TCSs can increase the placebo effect and impact the significance of the results; thus, a critical interpretation of the data is needed. Another consideration regards possible underdosing, especially for those therapies for which the dosage was adapted from the treatment of psoriasis, as was the case for ustekinumab. In addition, an accurate selection of patients based on disease severity can impact the magnitude of the effect induced with therapy (e.g., tezepelumab, fezakinumab). Finally, based on the main target of the drug, which can be AD clinical severity or specific AD symptoms, such as pruritus, an accurate elaboration of inclusion and exclusion criteria is crucial for the selection of the most suitable population. In fact, pruritus-targeted treatments appear to be most beneficial for mild AD (e.g., difelikefalin NCT04018027 and tradipitant NCT03568331), possibly due to the high, multipolar inflammatory burden characterizing moderate-to-severe AD. In this view, pruritus-related therapies are a valid option for mild or moderate AD or as combination therapy with other anti-inflammatory-directed treatments for more severe cases. From these experiences, it appears crucial to optimize the study design according to both the molecular targets and the clinical characteristics.

In conclusion, the combination of biomarkers and mechanistic studies with clinical efficacy in clinical trials helped select the most promising molecules and will shape the direction of future research. This translational revolution could lead to predicting patients’ responses to targeted therapy, thus guiding the choice of the most suitable treatment.

## References

[1] Hanifin JM, Reed ML, Eczema P, Impact Working G. (2007). A population-based survey of eczema prevalence in the United States. Dermatitis.

[2] Guttman-Yassky E, Nograles KE, Krueger JG (2011). Contrasting pathogenesis of atopic dermatitis and psoriasis-part I: clinical and pathologic concepts. J Allergy Clin Immunol.

[3] Lee HH, Patel KR, Singam V, Rastogi S, Silverberg JI (2019). A systematic review and meta-analysis of the prevalence and phenotype of adult-onset atopic dermatitis. J Am Acad Dermatol.

[4] Ellis CN, Mancini AJ, Paller AS, Simpson EL, Eichenfield LF (2012). Understanding and managing atopic dermatitis in adult patients. Semin Cutan Med Surg.

[5] Eichenfield LF, Tom WL, Chamlin SL, Feldman SR, Hanifin JM, Simpson EL (2014). Guidelines of care for the management of atopic dermatitis: section 1. Diagnosis and assessment of atopic dermatitis. J Am Acad Dermatol.

[6] Shaw TE, Currie GP, Koudelka CW, Simpson EL (2011). Eczema prevalence in the United States: data from the 2003 National Survey of Children’s Health. J Investig Dermatol.

[7] Kaufman BP, Guttman-Yassky E, Alexis AF (2018). Atopic dermatitis in diverse racial and ethnic groups-Variations in epidemiology, genetics, clinical presentation and treatment. Exp Dermatol.

[8] Williams HC, Pembroke AC, Forsdyke H, Boodoo G, Hay RJ, Burney PG (1995). London-born black Caribbean children are at increased risk of atopic dermatitis. J Am Acad Dermatol.

[9] Janumpally SR, Feldman SR, Gupta AK, Fleischer AB (2002). In the United States, blacks and Asian/Pacific Islanders are more likely than whites to seek medical care for atopic dermatitis. Arch Dermatol.

[10] Pesce G, Marcon A, Carosso A, Antonicelli L, Cazzoletti L, Ferrari M (2015). Adult eczema in Italy: prevalence and associations with environmental factors. J Eur Acad Dermatol Venereol.

[11] Silverberg JI, Gelfand JM, Margolis DJ, Boguniewicz M, Fonacier L, Grayson MH (2019). Pain Is a Common and Burdensome Symptom of Atopic Dermatitis in United States Adults. J Allergy Clin Immunol Pr.

[12] Silverberg JI, Gelfand JM, Margolis DJ, Boguniewicz M, Fonacier L, Grayson MH (2018). Patient burden and quality of life in atopic dermatitis in US adults: A population-based cross-sectional study. Ann Allergy Asthma Immunol.

[13] Simpson EL, Bieber T, Eckert L, Wu R, Ardeleanu M, Graham NM (2016). Patient burden of moderate to severe atopic dermatitis (AD): Insights from a phase 2b clinical trial of dupilumab in adults. J Am Acad Dermatol.

[14] Leung DY, Guttman-Yassky E (2014). Deciphering the complexities of atopic dermatitis: shifting paradigms in treatment approaches. J Allergy Clin Immunol.

[15] Leung DY (2000). Atopic dermatitis: new insights and opportunities for therapeutic intervention. J Allergy Clin Immunol.

[16] Baurecht H, Rühlemann MC, Rodríguez E, Thielking F, Harder I, Erkens AS (2018). Epidermal lipid composition, barrier integrity, and eczematous inflammation are associated with skin microbiome configuration. J Allergy Clin Immunol.

[17] Tsakok T, Woolf R, Smith CH, Weidinger S, Flohr C (2019). Atopic dermatitis: the skin barrier and beyond. Br J Dermatol.

[18] Nakatsuji T, Chen TH, Two AM, Chun KA, Narala S, Geha RS (2016). Staphylococcus aureus Exploits Epidermal Barrier Defects in Atopic Dermatitis to Trigger Cytokine Expression. J Investig Dermatol.

[19] Sidbury R, Davis DM, Cohen DE, Cordoro KM, Berger TG, Bergman JN (2014). Guidelines of care for the management of atopic dermatitis: section 3. Management and treatment with phototherapy and systemic agents. J Am Acad Dermatol.

[20] Aufiero BM, Talwar H, Young C, Krishnan M, Hatfield JS, Lee HK (2006). Narrow-band UVB induces apoptosis in human keratinocytes. J Photochem Photobio B.

[21] Hart PH, Gorman S, Finlay-Jones JJ (2011). Modulation of the immune system by UV radiation: more than just the effects of vitamin D?. Nat Rev Immunol.

[22] Jacobi A, Antoni C, Manger B, Schuler G, Hertl M (2005). Infliximab in the treatment of moderate to severe atopic dermatitis. J Am Acad Dermatol.

[23] Buka RL, Resh B, Roberts B, Cunningham BB, Friedlander S (2005). Etanercept is minimally effective in 2 children with atopic dermatitis. J Am Acad Dermatol.

[24] Uchida T, Suto H, Ra C, Ogawa H, Kobata T, Okumura K (2002). Preferential expression of T(h)2-type chemokine and its receptor in atopic dermatitis. Int Immunol.

[25] Gittler JK, Shemer A, Suarez-Farinas M, Fuentes-Duculan J, Gulewicz KJ, Wang CQ (2012). Progressive activation of T(H)2/T(H)22 cytokines and selective epidermal proteins characterizes acute and chronic atopic dermatitis. J Allergy Clin Immunol.

[26] Suárez-Fariñas M, Dhingra N, Gittler J, Shemer A, Cardinale I, de Guzman Strong C (2013). Intrinsic atopic dermatitis shows similar TH2 and higher TH17 immune activation compared with extrinsic atopic dermatitis. J Allergy Clin Immunol.

[27] Guttman-Yassky E, Lowes MA, Fuentes-Duculan J, Zaba LC, Cardinale I, Nograles KE (2008). Low expression of the IL-23/Th17 pathway in atopic dermatitis compared to psoriasis. J Immunol.

[28] Czarnowicki T, He H, Krueger JG, Guttman-Yassky E (2019). Atopic dermatitis endotypes and implications for targeted therapeutics. J Allergy Clin Immunol.

[29] Cabanillas B, Brehler AC, Novak N (2017). Atopic dermatitis phenotypes and the need for personalized medicine. Curr Opin Allergy Clin Immunol.

[30] Czarnowicki T, Esaki H, Gonzalez J, Malajian D, Shemer A, Noda S (2015). Early pediatric atopic dermatitis shows only a cutaneous lymphocyte antigen (CLA)(+) TH2/TH1 cell imbalance, whereas adults acquire CLA(+) TH22/TC22 cell subsets. J Allergy Clin Immunol.

[31] Esaki H, Brunner PM, Renert-Yuval Y, Czarnowicki T, Huynh T, Tran G (2016). Early-onset pediatric atopic dermatitis is T(H)2 but also T(H)17 polarized in skin. J Allergy Clin Immunol.

[32] Renert-Yuval Y, Del Duca E, Pavel AB, Fang M, Lefferdink R, Wu J (2021). The molecular features of normal and atopic dermatitis skin in infants, children, adolescents, and adults. J Allergy Clin Immunol.

[33] Noda S, Suárez-Fariñas M, Ungar B, Kim SJ, de Guzman Strong C, Xu H (2015). The Asian atopic dermatitis phenotype combines features of atopic dermatitis and psoriasis with increased TH17 polarization. J Allergy Clin Immunol.

[34] Chan TC, Sanyal RD, Pavel AB, Glickman J, Zheng X, Xu H (2018). Atopic dermatitis in Chinese patients shows T(H)2/T(H)17 skewing with psoriasiform features. J Allergy Clin Immunol.

[35] Sanyal RD, Pavel AB, Glickman J, Chan TC, Zheng X, Zhang N (2019). Atopic dermatitis in African American patients is T(H)2/T(H)22-skewed with T(H)1/T(H)17 attenuation. Ann Allergy Asthma Immunol.

[36] Brunner PM, Guttman-Yassky E (2019). Racial differences in atopic dermatitis. Ann Allergy Asthma Immunol.

[37] van der Velden VH, Laan MP, Baert MR, de Waal Malefyt R, Neijens HJ, Savelkoul HF (2001). Selective development of a strong Th2 cytokine profile in high-risk children who develop atopy: risk factors and regulatory role of IFN-gamma, IL-4 and IL-10. Clin Exp Allergy.

[38] Herberth G, Heinrich J, Röder S, Figl A, Weiss M, Diez U (2010). Reduced IFN-gamma- and enhanced IL-4-producing CD4+ cord blood T cells are associated with a higher risk for atopic dermatitis during the first 2 yr of life. Pediatr Allergy Immunol.

[39] Tang ML, Kemp AS, Thorburn J, Hill DJ (1994). Reduced interferon-gamma secretion in neonates and subsequent atopy. Lancet.

[40] Brunner PM, Israel A, Zhang N, Leonard A, Wen HC, Huynh T (2018). Early-onset pediatric atopic dermatitis is characterized by T(H)2/T(H)17/T(H)22-centered inflammation and lipid alterations. J Allergy Clin Immunol.

[41] Zhou L, Leonard A, Pavel AB, Malik K, Raja A, Glickman J (2019). Age-specific changes in the molecular phenotype of patients with moderate-to-severe atopic dermatitis. J Allergy Clin Immunol.

[42] Baum S, Porat S, Lyakhovitsky A, Astman N, Barzilai A (2019). Adult Atopic Dermatitis in Hospitalized Patients: Comparison between Those with Childhood-Onset and Late-Onset Disease. Dermatology.

[43] Karimkhani C, Silverberg JI, Dellavalle RP. Defining intrinsic vs. extrinsic atopic dermatitis. Dermatol Online J. 2015;21:13030/qt14p8p404. 26158358

[44] Tokura Y (2010). Extrinsic and intrinsic types of atopic dermatitis. J Dermatol Sci.

[45] Martel BC, Litman T, Hald A, Norsgaard H, Lovato P, Dyring-Andersen B (2016). Distinct molecular signatures of mild extrinsic and intrinsic atopic dermatitis. Exp Dermatol.

[46] Akdis CA, Akdis M (2003). Immunological differences between intrinsic and extrinsic types of atopic dermatitis. Clin Exp Allergy.

[47] Kulthanan K, Boochangkool K, Tuchinda P, Chularojanamontri L (2011). Clinical features of the extrinsic and intrinsic types of adult-onset atopic dermatitis. Asia Pac Allergy.

[48] Kabashima-Kubo R, Nakamura M, Sakabe J, Sugita K, Hino R, Mori T (2012). A group of atopic dermatitis without IgE elevation or barrier impairment shows a high Th1 frequency: possible immunological state of the intrinsic type. J Dermatol Sci.

[49] Kolls JK, McCray PB, Chan YR (2008). Cytokine-mediated regulation of antimicrobial proteins. Nat Rev Immunol.

[50] Park JH, Choi YL, Namkung JH, Kim WS, Lee JH, Park HJ (2006). Characteristics of extrinsic vs. intrinsic atopic dermatitis in infancy: correlations with laboratory variables. Br J Dermatol.

[51] Hamilton JD, Suárez-Fariñas M, Dhingra N, Cardinale I, Li X, Kostic A (2014). Dupilumab improves the molecular signature in skin of patients with moderate-to-severe atopic dermatitis. J Allergy Clin Immunol.

[52] Beck LA, Thaci D, Hamilton JD, Graham NM, Bieber T, Rocklin R (2014). Dupilumab treatment in adults with moderate-to-severe atopic dermatitis. N Engl J Med.

[53] Paller AS, Siegfried EC, Simpson EL, Cork MJ, Lockshin B, Kosloski MP (2021). A phase 2, open-label study of single-dose dupilumab in children aged 6 months to <6 years with severe uncontrolled atopic dermatitis: pharmacokinetics, safety and efficacy. J Eur Acad Dermatol Venereol.

[54] de Bruin-Weller M, Thaci D, Smith CH, Reich K, Cork MJ, Radin A (2018). Dupilumab with concomitant topical corticosteroid treatment in adults with atopic dermatitis with an inadequate response or intolerance to ciclosporin A or when this treatment is medically inadvisable: a placebo-controlled, randomized phase III clinical trial (LIBERTY AD CAFE). Br J Dermatol.

[55] Blauvelt A, de Bruin-Weller M, Gooderham M, Cather JC, Weisman J, Pariser D (2017). Long-term management of moderate-to-severe atopic dermatitis with dupilumab and concomitant topical corticosteroids (LIBERTY AD CHRONOS): a 1-year, randomised, double-blinded, placebo-controlled, phase 3 trial. Lancet.

[56] Simpson EL, Bieber T, Guttman-Yassky E, Beck LA, Blauvelt A, Cork MJ (2016). Two Phase 3 Trials of Dupilumab versus Placebo in Atopic Dermatitis. N Engl J Med.

[57] Nettis E, Ferrucci SM, Ortoncelli M, Pellacani G, Foti C, Di Leo E (2016). Use of Dupilumab in 543 Adult Patients With Moderate-to-Severe Atopic Dermatitis: A Multicenter, Retrospective Study. J Investig Allergol Clin Immunol.

[58] Wollenberg A, Blauvelt A, Guttman-Yassky E, Worm M, Lynde C, Lacour JP (2021). Tralokinumab for moderate-to-severe atopic dermatitis: results from two 52-week, randomized, double-blind, multicentre, placebo-controlled phase III trials (ECZTRA 1 and ECZTRA 2). Br J Dermatol.

[59] Silverberg JI, Toth D, Bieber T, Alexis AF, Elewski BE, Pink AE (2021). Tralokinumab plus topical corticosteroids for the treatment of moderate-to-severe atopic dermatitis: results from the double-blind, randomized, multicentre, placebo-controlled phase III ECZTRA 3 trial. Br J Dermatol.

[60] Guttman-Yassky E, Teixeira HD, Simpson EL, Papp KA, Pangan AL, Blauvelt A (2021). Once-daily upadacitinib versus placebo in adolescents and adults with moderate-to-severe atopic dermatitis (Measure Up 1 and Measure Up 2): results from two replicate double-blind, randomised controlled phase 3 trials. Lancet.

[61] Eichenfield LF, Flohr C, Sidbury R, Siegfried E, Szalai Z, Galus R (2021). Efficacy and Safety of Abrocitinib in Combination With Topical Therapy in Adolescents With Moderate-to-Severe Atopic Dermatitis: The JADE TEEN Randomized Clinical Trial. JAMA Dermatol.

[62] Simpson EL, Sinclair R, Forman S, Wollenberg A, Aschoff R, Cork M (2020). Efficacy and safety of abrocitinib in adults and adolescents with moderate-to-severe atopic dermatitis (JADE MONO-1): a multicentre, double-blind, randomised, placebo-controlled, phase 3 trial. Lancet.

[63] Reich K, Teixeira HD, de Bruin-Weller M, Bieber T, Soong W, Kabashima K (2021). Safety and efficacy of upadacitinib in combination with topical corticosteroids in adolescents and adults with moderate-to-severe atopic dermatitis (AD Up): results from a randomised, double-blind, placebo-controlled, phase 3 trial. Lancet.

[64] Roesner LM, Zeitvogel J, Heratizadeh A (2019). Common and different roles of IL-4 and IL-13 in skin allergy and clinical implications. Curr Opin Allergy Clin Immunol.

[65] Chan LS, Robinson N, Xu L (2001). Expression of interleukin-4 in the epidermis of transgenic mice results in a pruritic inflammatory skin disease: an experimental animal model to study atopic dermatitis. J Investig Dermatol.

[66] Zheng T, Oh MH, Oh SY, Schroeder JT, Glick AB, Zhu Z (2009). Transgenic expression of interleukin-13 in the skin induces a pruritic dermatitis and skin remodeling. J Investig Dermatol.

[67] Seneviratne SL, Jones L, Bailey AS, Black AP, Ogg GS (2006). Severe atopic dermatitis is associated with a reduced frequency of IL-10 producing allergen-specific CD4+ T cells. Clin Exp Dermatol.

[68] Kim BE, Leung DY, Boguniewicz M, Howell MD (2008). Loricrin and involucrin expression is down-regulated by Th2 cytokines through STAT-6. Clin Immunol.

[69] Howell MD, Kim BE, Gao P, Grant AV, Boguniewicz M, DeBenedetto A (2009). Cytokine modulation of atopic dermatitis filaggrin skin expression. J Allergy Clin Immunol.

[70] Metwally SS, Mosaad YM, Abdel-Samee ER, El-Gayyar MA, Abdel-Aziz AM, El-Chennawi FA (2004). IL-13 gene expression in patients with atopic dermatitis: relation to IgE level and to disease severity. Egypt J Immunol.

[71] Oh MH, Oh SY, Yu J, Myers AC, Leonard WJ, Liu YJ (2011). IL-13 induces skin fibrosis in atopic dermatitis by thymic stromal lymphopoietin. J Immunol.

[72] Biopharma C. Connect Biopharma Reports Detailed Positive Dataset from the Global Phase 2b Trial of CBP-201 in Adult Patients with Moderate-to-Severe Atopic Dermatitis https://www.biospace.com/article/releases/connect-biopharma-reports-detailed-positive-dataset-from-the-global-phase-2b-trial-of-cbp-201-in-adult-patients-with-moderate-to-severe-atopic-dermatitis/: Globe Newswire; 2022 [

[73] Popovic B, Breed J, Rees DG, Gardener MJ, Vinall LM, Kemp B (2017). Structural Characterisation Reveals Mechanism of IL-13-Neutralising Monoclonal Antibody Tralokinumab as Inhibition of Binding to IL-13Rα1 and IL-13Rα2. J Mol Biol.

[74] Gutermuth J, Pink AE, Worm M, Soldbro L, Bjerregard Oland C, Weidinger S (2022). Tralokinumab plus topical corticosteroids in adults with severe atopic dermatitis and inadequate response to or intolerance of ciclosporin A: a placebo-controlled, randomized, phase III clinical trial (ECZTRA 7). Br J Dermatol.

[75] Ultsch M, Bevers J, Nakamura G, Vandlen R, Kelley RF, Wu LC (2013). Structural basis of signaling blockade by anti-IL-13 antibody Lebrikizumab. J Mol Biol.

[76] Silverberg JI, Thaçi, D, Seneschal J, Stein Gold L, Blauvelt A, Simpson E, et al. Efficacy And Safety of Lebrikizumab in Moderate-to-Severe Atopic Dermatitis: Results from Two Phase 3, Randomized, Double-Blinded, Placebo-Controlled Trials. American Academy of Dermatology congress; Boston, MA, USA, March 15th–19th 2022. https://djbpnesxepydt.cloudfront.net/radv/April2022Abstracts/205-ADvocate-1---2-Primary-16-Wk_Silverberg-et-al_Abstract_1649532274839.pdf.

[77] Safety and Efficacy of Lebrikizumab (LY3650150) in Combination With Topical Corticosteroid in Moderate-to-Severe Atopic Dermatitis. (ADhere) https://clinicaltrials.gov/ct2/show/results/NCT04250337?term=NCT04250337&draw=2&rank=1 [

[78] Werfel T, Allam JP, Biedermann T, Eyerich K, Gilles S, Guttman-Yassky E (2016). Cellular and molecular immunologic mechanisms in patients with atopic dermatitis. J Allergy Clin Immunol.

[79] Kiehl P, Falkenberg K, Vogelbruch M, Kapp A (2001). Tissue eosinophilia in acute and chronic atopic dermatitis: a morphometric approach using quantitative image analysis of immunostaining. Br J Dermatol.

[80] Steigleder GK, Inderwisch R (1975). [Eosinophilic leucocytes in the skin lesions of psoriasis and atopic dermatitis (author’s transl)]. Arch Dermatol Res.

[81] Kägi MK, Joller-Jemelka H, Wüthrich B (1992). Correlation of eosinophils, eosinophil cationic protein and soluble interleukin-2 receptor with the clinical activity of atopic dermatitis. Dermatology.

[82] Simon D, Braathen LR, Simon HU (2004). Eosinophils and atopic dermatitis. Allergy.

[83] Leiferman KM (1994). Eosinophils in atopic dermatitis. J Allergy Clin Immunol.

[84] Kang EG, Narayana PK, Pouliquen IJ, Lopez MC, Ferreira-Cornwell MC, Getsy JA (2020). Efficacy and safety of mepolizumab administered subcutaneously for moderate to severe atopic dermatitis. Allergy.

[85] Oyoshi MK, Larson RP, Ziegler SF, Geha RS (2010). Mechanical injury polarizes skin dendritic cells to elicit a T(H)2 response by inducing cutaneous thymic stromal lymphopoietin expression. J Allergy Clin Immunol.

[86] Soumelis V, Reche PA, Kanzler H, Yuan W, Edward G, Homey B (2002). Human epithelial cells trigger dendritic cell mediated allergic inflammation by producing TSLP. Nat Immunol.

[87] Tidwell WJ, Fowler JF (2018). T-cell inhibitors for atopic dermatitis. J Am Acad Dermatol.

[88] Simpson EL, Parnes JR, She D, Crouch S, Rees W, Mo M (2019). Tezepelumab, an anti-thymic stromal lymphopoietin monoclonal antibody, in the treatment of moderate to severe atopic dermatitis: A randomized phase 2a clinical trial. J Am Acad Dermatol.

[89] Paterson DJ, Jefferies WA, Green JR, Brandon MR, Corthesy P, Puklavec M (1987). Antigens of activated rat T lymphocytes including a molecule of 50,000 Mr detected only on CD4 positive T blasts. Mol Immunol.

[90] Wang YH, Liu YJ (2009). Thymic stromal lymphopoietin, OX40-ligand, and interleukin-25 in allergic responses. Clin Exp Allergy.

[91] Czarnowicki T, Gonzalez J, Shemer A, Malajian D, Xu H, Zheng X (2015). Severe atopic dermatitis is characterized by selective expansion of circulating TH2/TC2 and TH22/TC22, but not TH17/TC17, cells within the skin-homing T-cell population. J Allergy Clin Immunol.

[92] Weinberg AD (1998). Antibodies to OX-40 (CD134) can identify and eliminate autoreactive T cells: implications for human autoimmune disease. Mol Med Today.

[93] Wang YH, Ito T, Wang YH, Homey B, Watanabe N, Martin R (2006). Maintenance and polarization of human TH2 central memory T cells by thymic stromal lymphopoietin-activated dendritic cells. Immunity.

[94] Guttman-Yassky E, Pavel AB, Zhou L, Estrada YD, Zhang N, Xu H (2019). GBR 830, an anti-OX40, improves skin gene signatures and clinical scores in patients with atopic dermatitis. J Allergy Clin Immunol.

[95] Sanofi. Press release: New, late-breaking data at EADV highlights emerging clinical profile of amlitelimab (formerly KY1005) in adults with inadequately controlled moderate-to-severe atopic dermatitis https://www.sanofi.com/en/media-room/press-releases/2021/2021-09-30-12-30-00-2306183September 30, 2021 [

[96] Weidinger S, et al. Treatment with amlitelimab-a novel non-depleting, non-cytotoxic anti-OX40Ligand monoclonal antibodyreduces IL-22 serum levels in a Phase 2a randomized, placebo-controlled trial in patients with moderate-to-severe atopic dermatitis. European Academy of Dermatology and Venereology congress; Milan, Italy, September 7th-11th 2022.

[97] Kirin K. Kyowa Kirin Announces Positive Phase 2 Results for KHK4083 in Patients with Moderate to Severe Atopic Dermatitis https://www.kyowakirin.com/media_center/news_releases/2021/e20210218_01.htmlFebruary 18, 2021.

[98] Nakagawa H, Iizuka H, Nemoto O, Shimabe M, Furukawa Y, Kikuta N (2020). Safety, tolerability and efficacy of repeated intravenous infusions of KHK4083, a fully human anti-OX40 monoclonal antibody, in Japanese patients with moderate to severe atopic dermatitis. J Dermatol Sci.

[99] Guttman-Yassky E et al. KHK4083/AMG451 (rocatinlimab), an anti-OX40 monoclonal antibody, provides durable transcriptomic improvement in skin of atopic dermatitis patients. European Academy of Dermatology and Venereology congress; Milan, Italy, September 7th-11th 2022.

[100] Zheng Y, Danilenko DM, Valdez P, Kasman I, Eastham-Anderson J, Wu J (2007). Interleukin-22, a T(H)17 cytokine, mediates IL-23-induced dermal inflammation and acanthosis. Nature.

[101] Gutowska-Owsiak D, Schaupp AL, Salimi M, Selvakumar TA, McPherson T, Taylor S (2012). IL-17 downregulates filaggrin and affects keratinocyte expression of genes associated with cellular adhesion. Exp Dermatol.

[102] Saeki H, Kabashima K, Tokura Y, Murata Y, Shiraishi A, Tamamura R (2017). Efficacy and safety of ustekinumab in Japanese patients with severe atopic dermatitis: a randomized, double-blind, placebo-controlled, phase II study. Br J Dermatol.

[103] Khattri S, Brunner PM, Garcet S, Finney R, Cohen SR, Oliva M (2017). Efficacy and safety of ustekinumab treatment in adults with moderate-to-severe atopic dermatitis. Exp Dermatol.

[104] A Study to Evaluate Risankizumab in Adults and Adolescents With Moderate to Severe Atopic Dermatitis https://clinicaltrials.gov/ct2/show/NCT03706040?term=risankizumab&cond=Atopic+Dermatitis&draw=2&rank=1

[105] Pan Y, Xu L, Qiao J, Fang H (2018). A systematic review of ustekinumab in the treatment of atopic dermatitis. J Dermatol Treat.

[106] Ungar B, Pavel AB, Li R, Kimmel G, Nia J, Hashim P (2021). Phase 2 randomized, double-blind study of IL-17 targeting with secukinumab in atopic dermatitis. J Allergy Clin Immunol.

[107] Nograles KE, Zaba LC, Shemer A, Fuentes-Duculan J, Cardinale I, Kikuchi T (2009). IL-22-producing “T22” T cells account for upregulated IL-22 in atopic dermatitis despite reduced IL-17-producing TH17 T cells. J Allergy Clin Immunol.

[108] Boniface K, Bernard FX, Garcia M, Gurney AL, Lecron JC, Morel F (2005). IL-22 inhibits epidermal differentiation and induces proinflammatory gene expression and migration of human keratinocytes. J Immunol.

[109] Gutowska-Owsiak D, Schaupp AL, Salimi M, Taylor S, Ogg GS (2011). Interleukin-22 downregulates filaggrin expression and affects expression of profilaggrin processing enzymes. Br J Dermatol.

[110] Eyerich S, Eyerich K, Pennino D, Carbone T, Nasorri F, Pallotta S (2009). Th22 cells represent a distinct human T cell subset involved in epidermal immunity and remodeling. J Clin Investig.

[111] Guttman-Yassky E, Brunner PM, Neumann AU, Khattri S, Pavel AB, Malik K (2018). Efficacy and safety of fezakinumab (an IL-22 monoclonal antibody) in adults with moderate-to-severe atopic dermatitis inadequately controlled by conventional treatments: A randomized, double-blind, phase 2a trial. J Am Acad Dermatol.

[112] Brunner PM, Pavel AB, Khattri S, Leonard A, Malik K, Rose S (2019). Baseline IL-22 expression in patients with atopic dermatitis stratifies tissue responses to fezakinumab. J Allergy Clin Immunol.

[113] Gu C, Wu L, Li X (2013). IL-17 family: cytokines, receptors and signaling. Cytokine.

[114] Guttman-Yassky E, Krueger JG (2018). IL-17C: A Unique Epithelial Cytokine with Potential for Targeting across the Spectrum of Atopic Dermatitis and Psoriasis. J Investig Dermatol.

[115] Ramirez-Carrozzi V, Sambandam A, Luis E, Lin Z, Jeet S, Lesch J (2011). IL-17C regulates the innate immune function of epithelial cells in an autocrine manner. Nat Immunol.

[116] Thaçi D, Singh D, Lee M, Timmis H, Jacobs D, Passier P (2011). Phase 1 and 2 Randomized Clinical Studies Determine Lack of Efficacy for Anti-IL-17C Antibody MOR106 in Moderate-Severe Atopic Dermatitis. J Clin Med.

[117] Galapagos. MOR106 clinical development in atopic dermatitis stopped for futility https://www.globenewswire.com/news-release/2019/10/28/1936632/0/en/MOR106-clinical-development-in-atopic-dermatitis-stopped-for-futility.html

[118] Abramovits W, Rivas Bejarano JJ, Valdecantos WC (2013). Role of interleukin 1 in atopic dermatitis. Dermatol Clin.

[119] Netea MG, Nold-Petry CA, Nold MF, Joosten LA, Opitz B, van der Meer JH (2009). Differential requirement for the activation of the inflammasome for processing and release of IL-1beta in monocytes and macrophages. Blood.

[120] Kupper TS, Ballard DW, Chua AO, McGuire JS, Flood PM, Horowitz MC (1986). Human keratinocytes contain mRNA indistinguishable from monocyte interleukin 1 alpha and beta mRNA. Keratinocyte epidermal cell-derived thymocyte-activating factor is identical to interleukin 1. J Exp Med.

[121] Yano S, Banno T, Walsh R, Blumenberg M (2008). Transcriptional responses of human epidermal keratinocytes to cytokine interleukin-1. J Cell Physiol.

[122] Mee JB, Johnson CM, Morar N, Burslem F, Groves RW (2007). The psoriatic transcriptome closely resembles that induced by interleukin-1 in cultured keratinocytes: dominance of innate immune responses in psoriasis. Am J Pathol.

[123] Weber A, Wasiliew P, Kracht M (2010). Interleukin-1 (IL-1) pathway. Sci Signal.

[124] Sims JE, Smith DE (2010). The IL-1 family: regulators of immunity. Nat Rev Immunol.

[125] Suárez-Fariñas M, Ungar B, Correa da Rosa J, Ewald DA, Rozenblit M, Gonzalez J (2015). RNA sequencing atopic dermatitis transcriptome profiling provides insights into novel disease mechanisms with potential therapeutic implications. J Allergy Clin Immunol.

[126] Foster AM, Baliwag J, Chen CS, Guzman AM, Stoll SW, Gudjonsson JE (2014). IL-36 promotes myeloid cell infiltration, activation, and inflammatory activity in skin. J Immunol.

[127] Gottlieb A. A Phase II, Open Label, Dose escalation study of bermekimab (MABp1) in patients with moderate to severe atopic dermatitis. European Academy of Dermatology and Venereology congress; Madrid, Spain, October 9th–13th 2019.

[128] ClinicalTrials.gov. A Study to Test the Long-term Safety of BI 655130 in Patients With Atopic Eczema Who Took Part in Study 1368-0032 https://www.clinicaltrials.gov/ct2/show/results/NCT04086121?term=NCT04086121&draw=2&rank=1

[129] Savinko T, Matikainen S, Saarialho-Kere U, Lehto M, Wang G, Lehtimaki S (2012). IL-33 and ST2 in atopic dermatitis: expression profiles and modulation by triggering factors. J Investig Dermatol.

[130] Murakami-Satsutani N, Ito T, Nakanishi T, Inagaki N, Tanaka A, Vien PT (2014). IL-33 promotes the induction and maintenance of Th2 immune responses by enhancing the function of OX40 ligand. Allergol Int.

[131] Miller AM (2011). Role of IL-33 in inflammation and disease. J Inflamm (Lond).

[132] Globe Newswire sA, Inc.. https://www.globenewswire.com/news-release/2019/11/08/1944020/0/en/AnaptysBio-Reports-Etokimab-ATLAS-Phase-2b-Clinical-Trial-in-Moderate-to-Severe-Atopic-Dermatitis-Fails-to-Meet-Primary-Endpoint.html Nov. 08, 2019

[133] ClinicalTrials.gov. Efficacy and Safety of REGN3500 Monotherapy and Combination of REGN3500 Plus Dupilumab in Adult Patients With Moderate-to-Severe Atopic Dermatitis. Accessed May 2022 https://clinicaltrials.gov/ct2/show/NCT03736967?term=NCT03736967&draw=2&rank=1

[134] Wechsler ME, Ruddy MK, Pavord ID, Israel E, Rabe KF, Ford LB (2021). Efficacy and Safety of Itepekimab in Patients with Moderate-to-Severe Asthma. N Engl J Med.

[135] He H, Guttman-Yassky E (2019). JAK Inhibitors for Atopic Dermatitis: An Update. Am J Clin Dermatol.

[136] Kontzias A, Kotlyar A, Laurence A, Changelian P, O’Shea JJ (2012). Jakinibs: a new class of kinase inhibitors in cancer and autoimmune disease. Curr Opin Pharm.

[137] Stritesky GL, Muthukrishnan R, Sehra S, Goswami R, Pham D, Travers J (2011). The transcription factor STAT3 is required for T helper 2 cell development. Immunity.

[138] Goswami R, Jabeen R, Yagi R, Pham D, Zhu J, Goenka S (2012). STAT6-dependent regulation of Th9 development. J Immunol.

[139] Tamura K, Arakawa H, Suzuki M, Kobayashi Y, Mochizuki H, Kato M (2001). Novel dinucleotide repeat polymorphism in the first exon of the STAT-6 gene is associated with allergic diseases. Clin Exp Allergy.

[140] Oetjen LK, Mack MR, Feng J, Whelan TM, Niu H, Guo CJ (2017). Sensory Neurons Co-opt Classical Immune Signaling Pathways to Mediate Chronic Itch. Cell.

[141] Fukuyama T, Ganchingco JR, Mishra SK, Olivry T, Rzagalinski I, Volmer DA (2017). Janus kinase inhibitors display broad anti-itch properties: A possible link through the TRPV1 receptor. J Allergy Clin Immunol.

[142] Wu NL, Huang DY, Tsou HN, Lin YC, Lin WW (2015). Syk mediates IL-17-induced CCL20 expression by targeting Act1-dependent K63-linked ubiquitination of TRAF6. J Investig Dermatol.

[143] Wu NL, Huang DY, Wang LF, Kannagi R, Fan YC, Lin WW (2016). Spleen Tyrosine Kinase Mediates EGFR Signaling to Regulate Keratinocyte Terminal Differentiation. J Investig Dermatol.

[144] Ackermann JA, Nys J, Schweighoffer E, McCleary S, Smithers N, Tybulewicz VL (2015). Syk tyrosine kinase is critical for B cell antibody responses and memory B cell survival. J Immunol.

[145] Whitney PG, Bär E, Osorio F, Rogers NC, Schraml BU, Deddouche S (2014). Syk signaling in dendritic cells orchestrates innate resistance to systemic fungal infection. PLoS Pathog.

[146] Alexis A, de Bruin-Weller M, Weidinger S, Soong W, Barbarot S, Ionita I (2022). Rapidity of Improvement in Signs/Symptoms of Moderate-to-Severe Atopic Dermatitis by Body Region with Abrocitinib in the Phase 3 JADE COMPARE Study. Dermatol Ther (Heidelb).

[147] Blauvelt A, Silverberg JI, Lynde CW, Bieber T, Eisman S, Zdybski J (2022). Abrocitinib induction, randomized withdrawal, and retreatment in patients with moderate-to-severe atopic dermatitis: Results from the JAK1 Atopic Dermatitis Efficacy and Safety (JADE) REGIMEN phase 3 trial. J Am Acad Dermatol.

[148] Blauvelt A, Teixeira HD, Simpson EL, Costanzo A, De Bruin-Weller M, Barbarot S (2021). Efficacy and Safety of Upadacitinib vs Dupilumab in Adults With Moderate-to-Severe Atopic Dermatitis: A Randomized Clinical Trial. JAMA Dermatol.

[149] Simpson EL, Forman S, Silverberg JI, Zirwas M, Maverakis E, Han G (2021). Baricitinib in patients with moderate-to-severe atopic dermatitis: Results from a randomized monotherapy phase 3 trial in the United States and Canada (BREEZE-AD5). J Am Acad Dermatol.

[150] Bissonnette R, Maari C, Forman S, Bhatia N, Lee M, Fowler J (2019). The oral Janus kinase/spleen tyrosine kinase inhibitor ASN002 demonstrates efficacy and improves associated systemic inflammation in patients with moderate-to-severe atopic dermatitis: results from a randomized double-blind placebo-controlled study. Br J Dermatol.

[151] Pavel AB, Song T, Kim HJ, Del Duca E, Krueger JG, Dubin C (2019). Oral Janus kinase/SYK inhibition (ASN002) suppresses inflammation and improves epidermal barrier markers in patients with atopic dermatitis. J Allergy Clin Immunol.

[152] Papp K, Szepietowski JC, Kircik L, Toth D, Eichenfield LF, Leung DYM (2021). Efficacy and safety of ruxolitinib cream for the treatment of atopic dermatitis: Results from 2 phase 3, randomized, double-blind studies. J Am Acad Dermatol.

[153] Bissonnette R, Papp KA, Poulin Y, Gooderham M, Raman M, Mallbris L (2016). Topical tofacitinib for atopic dermatitis: a phase IIa randomized trial. Br J Dermatol.

[154] ClinicalTrials.gov. A Study of ATI-502 Topical Solution for the Treatment of Atopic Dermatitis https://www.clinicaltrials.gov/ct2/show/results/NCT03585296?term=ATI-502&cond=Atopic+Dermatitis&draw=2&rank=1

[155] Furue M, Chiba T, Tsuji G, Ulzii D, Kido-Nakahara M, Nakahara T (2017). Atopic dermatitis: immune deviation, barrier dysfunction, IgE autoreactivity and new therapies. Allergol Int.

[156] Leung DY (1993). Role of IgE in atopic dermatitis. Curr Opin Immunol.

[157] Liu FT, Goodarzi H, Chen HY (2011). IgE, mast cells, and eosinophils in atopic dermatitis. Clin Rev Allergy Immunol.

[158] Heratizadeh A, Mittermann I, Balaji H, Wichmann K, Niebuhr M, Valenta R (2011). The role of T-cell reactivity towards the autoantigen alpha-NAC in atopic dermatitis. Br J Dermatol.

[159] Novak N, Bieber T, Kraft S (2004). Immunoglobulin E-bearing antigen-presenting cells in atopic dermatitis. Curr Allergy Asthma Rep..

[160] Wang HH, Li YC, Huang YC (2016). Efficacy of omalizumab in patients with atopic dermatitis: A systematic review and meta-analysis. J Allergy Clin Immunol.

[161] Chan S, Cornelius V, Cro S, Harper JI, Lack G (2020). Treatment Effect of Omalizumab on Severe Pediatric Atopic Dermatitis: The ADAPT Randomized Clinical Trial. JAMA Pediatr.

[162] Essayan DM (2001). Cyclic nucleotide phosphodiesterases. J Allergy Clin Immunol.

[163] Guttman-Yassky E, Hanifin JM, Boguniewicz M, Wollenberg A, Bissonnette R, Purohit V (2019). The role of phosphodiesterase 4 in the pathophysiology of atopic dermatitis and the perspective for its inhibition. Exp Dermatol.

[164] Abrahamsen H, Baillie G, Ngai J, Vang T, Nika K, Ruppelt A (2004). TCR- and CD28-mediated recruitment of phosphodiesterase 4 to lipid rafts potentiates TCR signaling. J Immunol.

[165] Simpson EL, Imafuku S, Poulin Y, Ungar B, Zhou L, Malik K (2019). A Phase 2 Randomized Trial of Apremilast in Patients with Atopic Dermatitis. J Investig Dermatol.

[166] Bissonnette R, Pavel AB, Diaz A, Werth JL, Zang C, Vranic I (2019). Crisaborole and atopic dermatitis skin biomarkers: An intrapatient randomized trial. J Allergy Clin Immunol.

[167] Paller AS, Tom WL, Lebwohl MG, Blumenthal RL, Boguniewicz M, Call RS (2016). Efficacy and safety of crisaborole ointment, a novel, nonsteroidal phosphodiesterase 4 (PDE4) inhibitor for the topical treatment of atopic dermatitis (AD) in children and adults. J Am Acad Dermatol.

[168] Esser C, Bargen I, Weighardt H, Haarmann-Stemmann T, Krutmann J (2013). Functions of the aryl hydrocarbon receptor in the skin. Semin Immunopathol.

[169] Furue M, Hashimoto-Hachiya A, Tsuji G (2019). Aryl Hydrocarbon Receptor in Atopic Dermatitis and Psoriasis. Int J Mol Sci.

[170] Furue M, Tsuji G, Mitoma C, Nakahara T, Chiba T, Morino-Koga S (2015). Gene regulation of filaggrin and other skin barrier proteins via aryl hydrocarbon receptor. J Dermatol Sci.

[171] Takei K, Mitoma C, Hashimoto-Hachiya A, Uchi H, Takahara M, Tsuji G (2015). Antioxidant soybean tar Glyteer rescues T-helper-mediated downregulation of filaggrin expression via aryl hydrocarbon receptor. J Dermatol.

[172] Funatake CJ, Marshall NB, Steppan LB, Mourich DV, Kerkvliet NI (2005). Cutting edge: activation of the aryl hydrocarbon receptor by 2,3,7,8-tetrachlorodibenzo-p-dioxin generates a population of CD4+ CD25+ cells with characteristics of regulatory T cells. J Immunol.

[173] Paller AS, Stein Gold L, Soung J, Tallman AM, Rubenstein DS, Gooderham M (2021). Efficacy and patient-reported outcomes from a phase 2b, randomized clinical trial of tapinarof cream for the treatment of adolescents and adults with atopic dermatitis. J Am Acad Dermatol.

[174] Ikoma A, Rukwied R, Stander S, Steinhoff M, Miyachi Y, Schmelz M (2003). Neuronal sensitization for histamine-induced itch in lesional skin of patients with atopic dermatitis. Arch Dermatol.

[175] Tani E, Shiosaka S, Sato M, Ishikawa T, Tohyama M (1990). Histamine acts directly on calcitonin gene-related peptide- and substance P-containing trigeminal ganglion neurons as assessed by calcium influx and immunocytochemistry. Neurosci Lett.

[176] Hofstra CL, Desai PJ, Thurmond RL, Fung-Leung WP (2003). Histamine H4 receptor mediates chemotaxis and calcium mobilization of mast cells. J Pharm Exp Ther.

[177] Dunford PJ, Williams KN, Desai PJ, Karlsson L, McQueen D, Thurmond RL (2007). Histamine H4 receptor antagonists are superior to traditional antihistamines in the attenuation of experimental pruritus. J Allergy Clin Immunol.

[178] Kim BM, Lee SH, Shim WS, Oh U (2004). Histamine-induced Ca(2+) influx via the PLA(2)/lipoxygenase/TRPV1 pathway in rat sensory neurons. Neurosci Lett.

[179] Suvas S (2017). Role of Substance P Neuropeptide in Inflammation, Wound Healing, and Tissue Homeostasis. J Immunol.

[180] Tominaga M, Ogawa H, Takamori K (2007). Possible Roles of Epidermal Opioid Systems in Pruritus of Atopic Dermatitis. J Investig Dermatol.

[181] Cheng B, Liu HW, Fu XB, Sheng ZY, Li JF (2008). Coexistence and upregulation of three types of opioid receptors, mu, delta and kappa, in human hypertrophic scars. Br J Dermatol.

[182] Salemi S, Aeschlimann A, Reisch N, Jüngel A, Gay RE, Heppner FL (2005). Detection of kappa and delta opioid receptors in skin—Outside the nervous system. Biochem Biophys Res Commun.

[183] Dillon SR, Sprecher C, Hammond A, Bilsborough J, Rosenfeld-Franklin M, Presnell SR (2004). Interleukin 31, a cytokine produced by activated T cells, induces dermatitis in mice. Nat Immunol.

[184] Sonkoly E, Muller A, Lauerma AI, Pivarcsi A, Soto H, Kemeny L (2006). IL-31: a new link between T cells and pruritus in atopic skin inflammation. J Allergy Clin Immunol.

[185] Kato A, Fujii E, Watanabe T, Takashima Y, Matsushita H, Furuhashi T (2014). Distribution of IL-31 and its receptor expressing cells in skin of atopic dermatitis. J Dermatol Sci.

[186] Tamura S, Morikawa Y, Miyajima A, Senba E (2003). Expression of oncostatin M receptor beta in a specific subset of nociceptive sensory neurons. Eur J Neurosci.

[187] Bando T, Morikawa Y, Komori T, Senba E (2006). Complete overlap of interleukin-31 receptor A and oncostatin M receptor beta in the adult dorsal root ganglia with distinct developmental expression patterns. Neuroscience.

[188] Cevikbas F, Wang X, Akiyama T, Kempkes C, Savinko T, Antal A (2014). A sensory neuron-expressed IL-31 receptor mediates T helper cell-dependent itch: Involvement of TRPV1 and TRPA1. J Allergy Clin Immunol.

[189] Kabashima K, Matsumura T, Komazaki H, Kawashima M, Nemolizumab JPSG (2020). Trial of Nemolizumab and Topical Agents for Atopic Dermatitis with Pruritus. N Engl J Med.

[190] kiniksa pharmaceuticals l. Kiniksa announces interim data from KPL-716 repeated-single-dose phase 1b clinical trial. https://www.globenewswire.com/August 12, 2019

[191] Tseng PY, Hoon MA (2021). Oncostatin M can sensitize sensory neurons in inflammatory pruritus. Sci Transl Med.

[192] Gazel A, Rosdy M, Bertino B, Tornier C, Sahuc F, Blumenberg M (2006). A characteristic subset of psoriasis-associated genes is induced by oncostatin-M in reconstituted epidermis. J Investig Dermatol.

[193] Werfel T, Layton G, Yeadon M, Whitlock L, Osterloh I, Jimenez P (2019). Efficacy and safety of the histamine H4 receptor antagonist ZPL-3893787 in patients with atopic dermatitis. J Allergy Clin Immunol.

[194] Welsh SE, Xiao C, Kaden AR, Brzezynski JL, Mohrman MA, Wang J (2021). Neurokinin-1 receptor antagonist tradipitant has mixed effects on itch in atopic dermatitis: results from EPIONE, a randomized clinical trial. J Eur Acad Dermatol Venereol.

[195] Kim B, Tamari M, Zamidar L, Nograles K, Munera C, Goncalves J. et al. Oral difelikefalin reduces pruritus in atopic dermatitis. European Academy of Dermatology and Venereology congress; virtual. Sep 29th-Oct 2nd 2021. https://www.caratherapeutics.com/wp-content/uploads/2022/05/Kim-B-et-al.-Presented-at-the-European-Academy-of-Dermatology-and-Venereology-Congress-2021.-1.pdf.

[196] Guttman-Yassky E, Facheris P, Correa Da Rosa J, Estrada Y, David E, Pavel A, et al. Oral difelikefalin improves itch and inflammatory biomarkers in atopic dermatitis subjects with moderate-to-severe pruritus. American Academy of Dermatology congress; Boston, USA, March 25th–28th 2022.

[197] Avila C, Massick S, Kaffenberger BH, Kwatra SG, Bechtel M (2020). Cannabinoids for the treatment of chronic pruritus: a review. J Am Acad Dermatol.

[198] Nam G, Jeong SK, Park BM, Lee SH, Kim HJ, Hong SP (2016). Selective Cannabinoid Receptor-1 Agonists Regulate Mast Cell Activation in an Oxazolone-Induced Atopic Dermatitis Model. Ann Dermatol.

[199] Schlosburg JE, O’Neal ST, Conrad DH, Lichtman AH (2011). CB1 receptors mediate rimonabant-induced pruritic responses in mice: investigation of locus of action. Psychopharmacol (Berl).

[200] Turcotte C, Blanchet MR, Laviolette M, Flamand N (2016). The CB(2) receptor and its role as a regulator of inflammation. Cell Mol Life Sci.

[201] Kim HJ, Kim B, Park BM, Jeon JE, Lee SH, Mann S (2015). Topical cannabinoid receptor 1 agonist attenuates the cutaneous inflammatory responses in oxazolone-induced atopic dermatitis model. Int J Dermatol.

[202] Small-Howard AL, Shimoda LM, Adra CN, Turner H (2005). Anti-inflammatory potential of CB1-mediated cAMP elevation in mast cells. Biochem J.

[203] Sugawara K, Bíró T, Tsuruta D, Tóth BI, Kromminga A, Zákány N (2012). Endocannabinoids limit excessive mast cell maturation and activation in human skin. J Allergy Clin Immunol.

[204] Muller C, Morales P, Reggio PH (2018). Cannabinoid Ligands Targeting TRP Channels. Front Mol Neurosci.

[205] Ambrosino P, Soldovieri MV, Russo C, Taglialatela M (2013). Activation and desensitization of TRPV1 channels in sensory neurons by the PPARα agonist palmitoylethanolamide. Br J Pharm.

[206] Eberlein B, Eicke C, Reinhardt HW, Ring J (2008). Adjuvant treatment of atopic eczema: assessment of an emollient containing N-palmitoylethanolamine (ATOPA study). J Eur Acad Dermatol Venereol.

[207] Pulvirenti N, Nasca MR, Micali G (2007). Topical adelmidrol 2% emulsion, a novel aliamide, in the treatment of mild atopic dermatitis in pediatric subjects: a pilot study. Acta Dermatovenerol Croat.

[208] Lee YW, Won CH, Jung K, Nam HJ, Choi G, Park YH (2019). Efficacy and safety of PAC-14028 cream - a novel, topical, nonsteroidal, selective TRPV1 antagonist in patients with mild-to-moderate atopic dermatitis: a phase IIb randomized trial. Br J Dermatol.

[209] Clausen ML, Edslev SM, Andersen PS, Clemmensen K, Krogfelt KA, Agner T (2017). Staphylococcus aureus colonization in atopic eczema and its association with filaggrin gene mutations. Br J Dermatol.

[210] Edslev SM, Agner T, Andersen PS (2020). Skin Microbiome in Atopic Dermatitis. Acta Derm Venereol.

[211] Kennedy EA, Connolly J, Hourihane JO, Fallon PG, McLean WHI, Murray D (2017). Skin microbiome before development of atopic dermatitis: Early colonization with commensal staphylococci at 2 months is associated with a lower risk of atopic dermatitis at 1 year. J Allergy Clin Immunol.

[212] Callewaert C, Nakatsuji T, Knight R, Kosciolek T, Vrbanac A, Kotol P (2020). IL-4Rα Blockade by Dupilumab Decreases Staphylococcus aureus Colonization and Increases Microbial Diversity in Atopic Dermatitis. J Investig Dermatol.

[213] Seite S, Flores GE, Henley JB, Martin R, Zelenkova H, Aguilar L (2014). Microbiome of affected and unaffected skin of patients with atopic dermatitis before and after emollient treatment. J Drugs Dermatol.

[214] Baldry M, Nakamura Y, Nakagawa S, Frees D, Matsue H, Núñez G (2018). Application of an agr-Specific Antivirulence Compound as Therapy for Staphylococcus aureus-Induced Inflammatory Skin Disease. J Infect Dis.

[215] Bath-Hextall FJ, Birnie AJ, Ravenscroft JC, Williams HC (2010). Interventions to reduce Staphylococcus aureus in the management of atopic eczema: an updated Cochrane review. Br J Dermatol.

[216] Nakatsuji T, Chen TH, Narala S, Chun KA, Two AM, Yun T (2017). Antimicrobials from human skin commensal bacteria protect against Staphylococcus aureus and are deficient in atopic dermatitis. Sci Transl Med.

[217] Cuello-Garcia CA, Brożek JL, Fiocchi A, Pawankar R, Yepes-Nuñez JJ, Terracciano L (2015). Probiotics for the prevention of allergy: A systematic review and meta-analysis of randomized controlled trials. J Allergy Clin Immunol.

[218] Shimamori Y, Pramono AK, Kitao T, Suzuki T, Aizawa S-I, Kubori T (2021). Isolation and Characterization of a Novel Phage SaGU1 that Infects Staphylococcus aureus Clinical Isolates from Patients with Atopic Dermatitis. Curr Microbiol.

[219] Kim SG, Giri SS, Yun S, Kim HJ, Kim SW, Kang JW (2020). Synergistic phage–surfactant combination clears IgE-promoted Staphylococcus aureus aggregation in vitro and enhances the effect in vivo. Int J Antimicrobial Agents.

[220] Pastagia M, Schuch R, Fischetti VA, Huang DB (2013). Lysins: the arrival of pathogen-directed anti-infectives. J Med Microbiol.

[221] Martin MJ, Estravís M, García-Sánchez A, Dávila I, Isidoro-García M, Sanz C (2011). Genetics and Epigenetics of Atopic Dermatitis: An Updated Systematic Review. Genes (Basel).

[222] Schmidt AD, de Guzman Strong C (2021). Current understanding of epigenetics in atopic dermatitis. Exp Dermatol.

[223] Rodríguez E, Baurecht H, Wahn AF, Kretschmer A, Hotze M, Zeilinger S (2014). An integrated epigenetic and transcriptomic analysis reveals distinct tissue-specific patterns of DNA methylation associated with atopic dermatitis. J Investig Dermatol.

[224] Acevedo N, Benfeitas R, Katayama S, Bruhn S, Andersson A, Wikberg G (2020). Epigenetic alterations in skin homing CD4(+)CLA(+) T cells of atopic dermatitis patients. Sci Rep..

[225] Bieber T (2020). Interleukin-13: Targeting an underestimated cytokine in atopic dermatitis. Allergy.

[226] Oh KS, Patel H, Gottschalk RA, Lee WS, Baek S, Fraser IDC (2017). Anti-Inflammatory Chromatinscape Suggests Alternative Mechanisms of Glucocorticoid Receptor Action. Immunity.

[227] ClinicalTrials.gov. Tralokinumab Monotherapy for Adolescent Subjects With Moderate to Severe Atopic Dermatitis - ECZTRA 6 (ECZema TRAlokinumab Trial no. 6) https://www.clinicaltrials.gov/ct2/show/results/NCT03526861?term=NCT03526861&draw=2&rank=1

[228] Gooderham MJ, Chu CY, Rojo R, Valdez H, Biswas P, Cameron MC (2021). Economic impact of abrocitinib monotherapy and combination therapy in patients with moderate-to-severe atopic dermatitis: results from JADE MONO-2 and JADE COMPARE. JAAD Int.

[229] Katoh N, Ohya Y, Murota H, Ikeda M, Hu X, Ikeda K (2022). A phase 3 randomized, multicenter, double-blind study to evaluate the safety of upadacitinib in combination with topical corticosteroids in adolescent and adult patients with moderate-to-severe atopic dermatitis in Japan (Rising Up): An interim 24-week analysis. JAAD Int.

[230] Simpson EL, Lacour JP, Spelman L, Galimberti R, Eichenfield LF, Bissonnette R (2020). Baricitinib in patients with moderate-to-severe atopic dermatitis and inadequate response to topical corticosteroids: results from two randomized monotherapy phase III trials. Br J Dermatol.

[231] Silverberg JI, Simpson EL, Wollenberg A, Bissonnette R, Kabashima K, DeLozier AM (2021). Long-term Efficacy of Baricitinib in Adults With Moderate to Severe Atopic Dermatitis Who Were Treatment Responders or Partial Responders: An Extension Study of 2 Randomized Clinical Trials. JAMA Dermatol.

[232] ClinicalTrials.gov. A Long-term Study of Baricitinib (LY3009104) With Topical Corticosteroids in Adults With Moderate to Severe Atopic Dermatitis That Are Not Controlled With Cyclosporine or for Those Who Cannot Take Oral Cyclosporine Because it is Not Medically Advisable (BREEZE-AD4) https://www.clinicaltrials.gov/ct2/show/results/NCT03428100?term=Baricitinib&cond=Atopic+Dermatitis&phase=2&draw=2&rank=6

[233] ClinicalTrials.gov. A Study of Baricitinib (LY3009104) in Participants With Moderate to Severe Atopic Dermatitis (BREEZE-AD6): https://www.clinicaltrials.gov/ct2/show/study/NCT03559270?term=Baricitinib&cond=Atopic+Dermatitis&phase=2&draw=2&rank=1

[234] Reich K, Kabashima K, Peris K, Silverberg JI, Eichenfield LF, Bieber T (2020). Efficacy and Safety of Baricitinib Combined With Topical Corticosteroids for Treatment of Moderate to Severe Atopic Dermatitis: A Randomized Clinical Trial. JAMA Dermatol.

[235] ClinicalTrials.gov. Phase 2B Study to Evaluate ASN002 in Subjects With Moderate to Severe Atopic Dermatitis (RADIANT) https://www.clinicaltrials.gov/ct2/show/NCT03531957?term=asn002&cond=Atopic+Dermatitis&draw=2&rank=2

[236] ClinicalTrials.gov. Study to Evaluate Long-Term Safety of ASN002 in Subjects With Moderate to Severe Atopic Dermatitis https://www.clinicaltrials.gov/ct2/show/NCT03654755?term=asn002&cond=Atopic+Dermatitis&draw=2&rank=1

[237] Samrao A, Berry TM, Goreshi R, Simpson EL (2012). A pilot study of an oral phosphodiesterase inhibitor (apremilast) for atopic dermatitis in adults. Arch Dermatol.

[238] Volf EM, Au SC, Dumont N, Scheinman P, Gottlieb AB (2012). A phase 2, open-label, investigator-initiated study to evaluate the safety and efficacy of apremilast in subjects with recalcitrant allergic contact or atopic dermatitis. J Drugs Dermatol.

[239] ClinicalTrials.gov. An Investigator-initiated Study of Apremilast to Demonstrate Efficacy Nummular Eczema (APREMINUM) https://www.clinicaltrials.gov/ct2/show/NCT03160248?term=apremilast&cond=Atopic+Dermatitis&draw=2&rank=5

[240] ClinicalTrials.gov. Efficacy and Safety Study of QAW039 in the Treatment of Patients With Moderate to Severe Atopic Dermatitis. https://www.clinicaltrials.gov/ct2/show/NCT01785602?term=NCT01785602&draw=2&rank=1

[241] ClinicalTrials.gov. Effect of OC000459 on Moderate to Severe Atopic Dermatitis https://www.clinicaltrials.gov/ct2/show/NCT02002208?term=OC000459&cond=Atopic+Dermatitis&draw=2&rank=1

[242] ClinicalTrials.gov. Efficacy and Safety of Orally Administered DS107 in Adult Patients With Moderate to Severe Atopic Dermatitis https://www.clinicaltrials.gov/ct2/show/NCT03817190?term=DS107&cond=Atopic+Dermatitis&phase=1&draw=2&rank=1

[243] Pharmaceuticals V. Vanda Pharmaceuticals Announces Tradipitant Phase II Proof of Concept Study Results for Chronic Pruritus in Atopic Dermatitis https://www.prnewswire.com/news-releases/vanda-pharmaceuticals-announces-tradipitant-phase-ii-proof-of-concept-study-results-for-chronic-pruritus-in-atopic-dermatitis-300045700.html: PR Newswire; 2015

[244] ClinicalTrials.gov. Tradipitant in Treatment-resistant Pruritus Associated With Atopic Dermatitis: https://www.clinicaltrials.gov/ct2/show/NCT02651714?term=tradipitant&cond=Atopic+Dermatitis&draw=2&rank=1

[245] ClinicalTrials.gov. Study of the Efficacy, Safety, and Tolerability of Serlopitant for Pruritus (Itch) in Atopic Dermatitis (ATOMIK) https://www.clinicaltrials.gov/ct2/show/NCT02975206?term=serlopitant&cond=Atopic+Dermatitis&draw=2&rank=1

[246] ClinicalTrials.gov. Dose-ranging Trial to Evaluate Delgocitinib Cream 1, 3, 8, and 20 mg/g Compared to Delgocitinib Cream Vehicle Over an 8-week Treatment Period in Adult Subjects With Atopic Dermatitis. https://www.clinicaltrials.gov/ct2/show/NCT03725722?term=DELGoCITINIB&cond=Atopic+Dermatitis&phase=12&draw=2&rank=1

[247] Nakagawa H, Nemoto O, Igarashi A, Saeki H, Kaino H, Nagata T (2020). Delgocitinib ointment, a topical Janus kinase inhibitor, in adult patients with moderate to severe atopic dermatitis: A phase 3, randomized, double-blind, vehicle-controlled study and an open-label, long-term extension study. J Am Acad Dermatol.

[248] Sienna. Sienna Biopharmaceuticals announces results from firstin-human study of SNA-125 in psoriasis and continued progression to phase 2 [press release]. https://www.globenewswire.com/news-release/2018/12/03/1660673/0/en/Sienna-Biopharmaceuticals-Announces-Its-Topical-Non-Steroidal-TrkA-Inhibitor-SNA-120-0-05-Demonstrated-Significant-Impact-on-Psoriasis-in-Phase-2b-Study-and-Plans-to-Initiate-Phase.html 2018 Aug 27

[249] ClinicalTrials.gov. Crisaborole for Chinese and Japanese Subjects (≥2 Years of Age) With Mild to Moderate Atopic Dermatitis: https://www.clinicaltrials.gov/ct2/show/NCT04360187?term=crisaborole&cond=Atopic+Dermatitis&phase=2&draw=2&rank=3

[250] ClinicalTrials.gov. A Study of the Long-Term Safety of Crisaborole Ointment, 2% in Japanese Pediatric and Adult Participants With Mild to Moderate Atopic Dermatitis https://www.clinicaltrials.gov/ct2/show/NCT04498403?term=crisaborole&cond=Atopic+Dermatitis&phase=2&draw=2&rank=1

[251] Saeki H, Ito K, Yokota D, Tsubouchi H (2022). Difamilast ointment in adult patients with atopic dermatitis: a phase 3 randomized, double-blind, vehicle-controlled trial. J Am Acad Dermatol.

[252] Saeki H, Baba N, Ito K, Yokota D, Tsubouchi H (2022). Difamilast, a selective phosphodiesterase 4 inhibitor, ointment in paediatric patients with atopic dermatitis: a phase III randomized double-blind, vehicle-controlled trial. Br J Dermatol.

[253] ClinicalTrials.gov. Long-term Trial of OPA-15406 Ointment in Adult and Pediatric Patients With Atopic Dermatitis https://www.clinicaltrials.gov/ct2/show/NCT03961529?term=NCT03961529&cond=Atopic+Dermatitis&draw=2&rank=1

[254] ClinicalTrials.gov. Topical Roflumilast in Adults With Atopic Dermatitis https://www.clinicaltrials.gov/ct2/show/NCT01856764?term=NCT01856764&draw=2&rank=1

[255] Furue M, Kitahara Y, Akama H, Hojo S, Hayashi N, Nakagawa H (2014). Safety and efficacy of topical E6005, a phosphodiesterase 4 inhibitor, in Japanese adult patients with atopic dermatitis: results of a randomized, vehicle-controlled, multicenter clinical trial. J Dermatol.

[256] ClinicalTrials.gov. A Study to Determine the Safety & Efficacy of ZPL-5212372 in Healthy Subjects and in Subjects With Atopic Dermatitis https://clinicaltrials.gov/ct2/show/NCT02795832?term=ZPL-5212372&draw=2&rank=1

[257] Niemeyer-van der Kolk T, van der Wall H, Hogendoorn GK, Rijneveld R, Luijten S, van Alewijk D (2020). Pharmacodynamic Effects of Topical Omiganan in Patients With Mild to Moderate Atopic Dermatitis in a Randomized, Placebo-Controlled, Phase II Trial. Clin Transl Sci.

[258] Niemeyer-van der Kolk T, Buters TP, Krouwels L, Boltjes J, de Kam ML, van der Wall H (2022). Topical antimicrobial peptide omiganan recovers cutaneous dysbiosis but does not improve clinical symptoms in patients with mild to moderate atopic dermatitis in a phase 2 randomized controlled trial. J Am Acad Dermatol.

[259] Czarnowicki T, Dohlman AB, Malik K, Antonini D, Bissonnette R, Chan TC (2018). Effect of short-term liver X receptor activation on epidermal barrier features in mild to moderate atopic dermatitis: a randomized controlled trial. Ann Allergy Asthma Immunol.

